# A Drug‐Gated, Modular STAb‐T Immunotherapy With External Control

**DOI:** 10.1002/advs.77236

**Published:** 2026-08-24

**Authors:** Susana Luengo‐Arias, Carmen Domínguez‐Alonso, Ivana Zagorac, Antonio Tapia‐Galisteo, Eva García‐Veros, Laura Rubio‐Pérez, Marina Gómez‐Rosel, Patricia Fuentes, Patricia Hernández‐López, Óscar Aguilar‐Sopeña, Laura Almagro‐Puente, Sonia Martínez‐González, Lucía Cañizares‐Moscato, María Rivas‐Sánchez, Belén Blanco, Ángel Ramírez‐Fernández, Laura Díez‐Alonso, Carmen Blanco‐Aparicio, Javier Arroyo‐Ródenas, Jorge Martínez‐Torrecuadrada, María L. Toribio, Pedro Roda‐Navarro, Anaïs Jiménez‐Reinoso, Luis Álvarez‐Vallina

**Affiliations:** ^1^ Cancer Immunotherapy Unit (UNICA) Department of Immunology Hospital Universitario 12 de Octubre Madrid Spain; ^2^ Immuno‐Oncology and Immunotherapy Group Instituto de Investigación Sanitaria Hospital 12 de Octubre (imas12) Madrid Spain; ^3^ CNIO‐HMRIB Cancer Immunotherapy Clinical Research Unit Spanish National Cancer Research Centre (CNIO) Madrid Spain; ^4^ Centro de Biología Molecular (CBM) Severo Ochoa CSIC‐UAM Madrid Spain; ^5^ Department of Immunology Ophthalmology and ENT School of Medicine Universidad Complutense Madrid Spain; ^6^ Lymphocyte Immunobiology Group Instituto De Investigación Sanitaria 12 De Octubre (imas12) Madrid Spain; ^7^ Experimental Therapeutics Programme Spanish National Cancer Research Centre (CNIO) Madrid Spain; ^8^ Protein Production Unit Spanish National Cancer Research Center (CNIO) Madrid Spain; ^9^ Department of Advanced Therapy Drug Development Instituto De Salud Carlos III Madrid Spain; ^10^ Cancer Immunotherapy Hospital Del Mar Research Institute Barcelona (HMRIB) Barcelona Spain; ^11^ Advanced Therapies Program Hospital Del Mar Research Institute Barcelona (HMRIB) Barcelona Spain; ^12^ Banc de Sang i Teixits Barcelona Spain

**Keywords:** hematological malignancies, rapalog, STAb, switchable TCE, T cell engagers

## Abstract

Living cell therapies lack robust, reversible mechanisms for externally controlling therapeutic activity after administration, limiting their safety and clinical adaptability. Here we engineer a drug‐gated cellular immunotherapy platform in which T cells function as programmable factories that secrete two inactive antibody modules whose extracellular assembly into a functional bispecific T cell engager (TCE) is controlled by a small‐molecule input. Using a rapalog‐inducible FKBP‐FRB* heterodimerization switch, we design a split CD19 × CD3 engager architecture that remains inactive in the absence of drug and assembles on demand upon rapalog exposure. A 2A‐peptide bicistronic construct enables coordinated expression and secretion of both modules, allowing precise drug‐dependent control of TCE formation in situ. Drug administration quantitatively regulates T cell activation and cytotoxicity against CD19^+^ targets in vitro, with stringent OFF‐state behavior in the absence of rapalog. In xenograft models, systemic rapalog administration induces on‐demand anti‐tumor activity without evidence of treatment‐related toxicity, demonstrating reversible pharmacological control of a locally secreted therapeutic interface. We further extend this strategy to an EGFR‐targeting TCE, demonstrating the modularity and broad adaptability of the platform across distinct antigen specificities. This work introduces a generalizable engineering framework for externally programmable cell therapies, enabling tunable, safety‐by‐design control of T cell‐based immunotherapies.

## Graphical abstract

Engineered STAb‐T^ON^ cells act as programmable living factories that continuously secrete inactive antibody modules, which assemble extracellularly into a functional T cell engager only upon small‐molecule administration. This externally controlled platform enables reversible, on‐demand regulation of antitumor immunity, establishing a new paradigm for safer and programmable cell therapies. Created in https://BioRender.com.

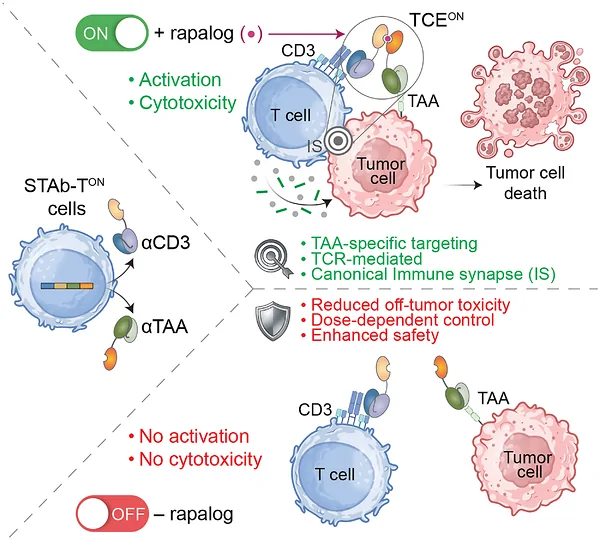

**Abbreviations**
2Tx: Double transfected HEK293 cells (CD19‐FKBP+CD3‐FRB*)
Blue CMAC: 7‐amino‐4‐Chloromethylcoumarin
CAR: Chimeric antigen receptor
CM: Conditioned media
CRS: Cytokine release syndrome
DCM: DMEM complete medium
E:T: Effector:target
EGFR: Epidermal growth factor receptor
FITC: Fluorescein isothiocyanate
FKBP: FK506 binding protein
FRB*: FKBP rapamycin binding domain (T2089L)
F/S: FLAG and Strep‐tagII epitopes
GFP: Green fluorescent protein
G‐418: Geneticin
HEK293T^ON^
: HEK293T cells secreting TCE^ON^
HPLC: High‐performance liquid chromatography
HRP: Horseradish peroxidase
IFN‐γ: Interferon‐γ
IL: Interleukin
IMAC: Immobilized‐metal affinity chromatography
i.p.: Intraperitoneally
IS: Immunological synapse
J‐NT: Non‐transduced Jurkat T cells
J‐STAb‐T^C^
: Jurkat STAb‐T cells secreting TCE^C^
J‐STAb‐T^ON^
: Jurkat STAb‐T cells secreting TCE^ON^
KO: Knock‐out
LLOQ: Lower limit of quantification
Luc: Firefly luciferase gene
mAb: Monoclonal antibodies
MFI: Median fluorescence intensity
MS: Mass spectrometry
MOI: Multiplicities of infection
NT‐T: Non‐transduced primary T cells
NTx: Non‐transfected cells
NSG: NOD.Cγ‐*Prkdc^scid^ Il2rg^1Wjl^*/SzJ mice female mice
NXG: NOD‐Prkdc^scid^‐IL2rg^1^/Rj female mice
OM: Oncostatin M
PBMC: Peripheral blood mononuclear cells
PROTAC: Proteolysis‐targeted chimera
pTyr: Phospho‐tyrosine
RCM: RPMI complete medium
Rapalog: Rapamycin analog
s.c.: Subcutaneously
scFv: Single‐chain variable fragment
SD: Standard deviation
SEC: Size‐exclusion chromatography
SEE: Staphylococcal enterotoxin E
SEM: Standard error of the mean
STAb: Secreting T cell‐engaging antibodies
STAb‐T^C^
: STAb‐T cells secreting TCE^C^
STAb‐T^ON^
: STAb‐T cells secreting TCE^ON^
TAA: Tumor‐associated antigens
TCEs: T cell engagers
TCE^C^
: Conventional anti‐CD19 TCE
TCE^ON^
: ON‐switch anti‐CD19 TCE
T_CM_
: Central memory T cells
T_EF_
: Effector T cells
T_EMRA_
: Effector memory T cells expressing CD45RA
tdTo: tdTomato fluorescent protein
TMB: Tetramethylbenzidine
T_N_
: Naïve T cells
UV: Ultraviolet
V_H_
: Variable heavy
V_L_
: Variable light

## 1 Introduction

Strategies that redirect T cells to recognize cell surface tumor‐associated antigens (TAAs) are transforming cancer therapy paradigms [1]. However, from a biomedical engineering perspective, a central limitation of current T cell‐redirecting therapies is the lack of precise, reversible, and externally addressable control over therapeutic activity once cells or biologics are administered. T cell redirection has primarily been achieved through two approaches: the adoptive transfer of T cells engineered to express chimeric antigen receptors (CARs), and the systemic administration of bispecific T cell‐engaging (TCE) antibodies, which simultaneously binds a TAA on tumor cells and the CD3ε chain of the TCR/CD3 complex on T cells [2, 3]. An emerging approach, termed STAb‐T (secreting T cell‐engaging antibodies), involves the in situ secretion of TCEs by genetically modified T cells [4, 5, 6, 7, 8]. This strategy aims to harness the tumor‐homing capacity of adoptively transferred T cells, while enabling local recruitment of endogenous polyclonal T cells by secreting TCE into the tumor [2, 4, 9, 10, 11, 12]. These strategies operate largely as constitutively active systems, offering limited opportunities for dynamic modulation of therapeutic output in vivo.

T cell‐redirecting therapies are associated with significant adverse effects, including cytokine release syndrome (CRS) and neurotoxicity [13]. These challenges are further exacerbated in the context of solid tumors, where most TAAs are also expressed on essential healthy tissues, resulting in on‐target/off‐tumor toxicity [13]. From an engineering standpoint, these toxicities reflect insufficient control over the timing, intensity, and spatial distribution of T cell activation. While the dose of TCEs can be modulated to mitigate such effects, adoptively transferred CAR‐T cells are less amenable to fine control [14, 15]. To address these safety concerns, several strategies have been proposed to eliminate infused T cells upon detection of toxicity, including the incorporation of suicide genes [16, 17] and antibody‐mediated depletion of engineered T cells [18, 19]. However, these approaches typically result in the irreversible loss of a highly personalized, costly, and time‐consuming therapeutic product, often before any therapeutic benefit can be realized [20, 21]. Consequently, the development of controllable and reversible strategies to modulate engineered T cell activity could significantly advance their clinical translation.

Several controllable platforms have been developed to serve as reversible safety switches for CAR‐T cell function, including inducible gene expression systems [22, 23, 24], drug‐induced heterodimerization [25, 26], proteolysis‐targeted chimera (PROTAC) technology [27], and split CAR constructs, in which the antigen recognition and signaling domains are engineered with heterodimerizing motifs that assemble only in the presence of an exogenous factor, such as a small molecule [25, 28, 29, 30, 31]. Small molecule‐based regulation offers the advantages of improved tissue penetration and rapid pharmacokinetics due to fast systemic clearance [32]. Split CAR designs separate antigen binding and signaling domains, allowing functional control by administration of the dimerizing component required for receptor assembly. Universal CAR approaches use a different strategy in which T cells express a CAR that does not directly recognize a TAA but instead binds to an adaptor molecule. These T cells remain inactive until the adaptor, fused to an anti‐TAA moiety, is administered [21]. In this system, the anti‐TAA adaptor acts as a molecular switch, activating CAR‐T cells when it is present and dampening activity when it is absent. Various adaptors have been explored for this purpose, including biotin [33, 34], fluorescein isothiocyanate (FITC) [35, 36], leucine zipper domains [37], mutated forms of NKG2D [38], and engineered neoepitopes [20, 39, 40]. While modular CAR systems offer flexible and tunable antigen targeting, they often require continuous administration of the antigen recognition module, increasing therapeutic complexity and cost [21].

In the context of systemically administered TCEs, a modular system consisting of two components has been introduced: a target domain consisting of an anti‐TAA single‐chain variable fragment (scFv) fused to a 10‐amino‐acid peptide epitope (E5B9), and an effector domain consisting of a bispecific tandem scFv targeting CD3 and E5B9 [41]. More recently, a trispecific antibody system has been developed in which two separate modules—each containing a scFv against a different TAA—are fused to either the variable heavy (V_H_) or variable light (V_L_) chain of an anti‐CD3 domain [42]. Upon binding to their respective TAAs on the same target cell, the V_H_ and V_L_ chains colocalize, enabling reconstitution of the functional anti‐CD3 binding site and subsequent T cell redirection. While these modular strategies enhance target specificity, they still share key limitations with conventional TCEs, including the need for systemic and continuous infusion to maintain therapeutic efficacy [1, 2].

Here, we report a drug‐gated STAb‐T cell platform that introduces an external molecular control layer over therapeutic assembly. We describe the first generation of switchable STAb‐T cells based on a split TCE system for the treatment of CD19^+^ hematological malignancies. Assembly of the functional TCE is governed by a molecular ON‐switch relying on heterodimerization between FK506 binding protein (FKBP) and a mutant FKBP–rapamycin binding domain (FRB*; T2089L) in the presence of the rapamycin analog AP21967 (rapalog). Engineered T cells secrete both inactive TCE modules, but formation of the functional anti‐CD19 × anti‐CD3 engager occurs only upon rapalog administration. This architecture enables reversible, dose‐dependent, and externally programmable control of T cell activation and cytotoxicity. To demonstrate the broader applicability of this approach, we further engineered an EGFR‐targeting TCE using the same split architecture, establishing the modularity of the platform across distinct antigen specificities.

## 2 Materials and Methods

### 2.1 Bacterial Cells and Culture Conditions

*Top10* or *Stbl3 E. coli* strains were used for plasmid propagation and cloning following standard chemically competent transformation protocols. Bacteria were grown in LB‐agar plates or 2xTY medium (both from Carl Roth GmbH, Karlsruhe, Germany), and ampicillin (Merck Millipore, Tullagreen, Carrigtwohill, Ireland) at 100 µg/mL was used as a selection antibiotic.

### 2.2 Mammalian Cells and Culture Conditions

All tumor cell lines were purchased from either the American Type Culture Collection (Rockville, MD, USA) or the DSMZ (Braunschweig, Germany). HEK293 (CRL‐1573), HEK293T (CRL‐3216), CHO (CCL‐61), HCT116 (CCL‐247) and A549 (CCL‐185) cells were cultured in DMEM (Lonza, Walkersville, MD, USA) supplemented with 10% (v/v) heat inactivated fetal bovine serum (FBS; Sigma‐Aldrich, Burlington, MA, USA), 2 mM L‐glutamine (Life Technologies, Paisley, UK) and antibiotics (100 units/mL penicillin, 100 µg/mL streptomycin) (both from Sigma‐Aldrich), hereafter referred to from here on as DMEM complete medium (DCM). Jurkat clone E6‐1 (TIB‐152), K562 (CCL‐243), Raji (CCL‐86), and NALM6 (CRL‐3273) were cultured in RPMI‐1640 (Lonza) supplemented with 10% (v/v) heat inactivated FBS, 2 mM L‐glutamine and antibiotics referred to as RPMI complete medium (RCM). NALM6, K562 and SEM cells expressing the firefly luciferase (Luc) gene (NALM6^Luc^, K562^Luc^, SEM^Luc^, CHO^Luc^ and HCT116^Luc^) were generated as previously described [43, 44]. SEM cells (SEM^WT^, CVCL_0095) and SEM CD19 knock‐out (KO) cells (SEM^CD19KO^) [45] were cultured in IMDM (Lonza) supplemented with 10% (v/v) heat‐inactivated FBS, 2 mM L‐glutamine, antibiotics and 25 mM Hepes (Lonza). All the cell lines were grown at 37°C in 5% CO_2_ and routinely screened for mycoplasma contamination by PCR (Mycoplasma Gel Detection Kit; Biotools, Madrid, Spain).

### 2.3 Mouse Models

Mice were housed under pathogen‐free conditions in the animal facility of the Centro Nacional de Investigaciones Oncológicas (CNIO, Madrid, Spain), where in vivo experiments were performed in accordance with the European Union Directive 2010/63/UE, transposed into Spanish law under RD 53/2013. All animal procedures were approved by the Animal Ethics Committee of the Instituto de Salud Carlos III and were performed in accordance with the guidelines of the International Guiding Principles for Biomedical Research Involving Animals, established by the Council for International Organizations of Medical Sciences. The experimental study protocols were also approved by the Comunidad de Madrid (PROEX 272.2/23). Eight‐to‐twelve weeks old NOD.Cg‐*Prkdc^scid^ Il2rg^1Wjl^*/SzJ female mice (NSG, The Jackson Laboratory, ME, USA) were purchased for the short‐term in vivo experiment. Six‐to‐eight weeks old NOD‐Prkdc^scid^‐IL2rg^1^/Rj female mice (NXG, JANVIER LABS, Le Genest‐Saint‐Isle, France) were purchased for the medium‐term in vivo experiment.

### 2.4 Construction of Expression Vectors

The expression vector pCDNA3.1‐A3B1‐OKT3 encoding the human kappa light chain signal peptide L1, the anti‐CD19 A3B1 scFv (V_L_–V_H_), a five‐residue linker (G_4_S), the anti‐CD3 OKT3 scFv (V_H_–V_L_) [46], and a C‐terminal polyHis has been described previously [4]. Synthetic genes encoding the FKBP and FRB* domains [31] were synthesized by GeneArt AG (ThermoFisher Scientific, Regensburg, Germany). The FKBP domain was cloned into the pCR3.1 plasmid by *Not*I*/Xba*I digestion, followed by the oncostatin M (OM) signal peptide, FLAG and Strep‐tagII epitopes (F/S), and the A3B1 scFv (V_L_–V_H_) (CD19‐FKBP). The FRB* domain followed by a C‐terminal polyHis (His) tag was cloned into the pCR3.1 plasmid by *Not*I*/Bam*HI digestion after the OM signal peptide and the OKT3 scFv (V_H_‐V_L_) (CD3‐FRB*). The OM‐OKT3‐FRB*‐H construct was further subcloned by *Hind*III*/Xba*I digestion into the pCDNA3.1 hygro+ plasmid (Thermo Fisher Scientific, V79520). The OM‐F/S‐A3B1‐FKBP and OM‐OKT3‐FRB*‐His constructs were inserted by *Bam*HI*/Xba*I and *Hind*III*/Bgl*II digestion, respectively, into the pCR3.1 plasmid containing a foot‐and‐mouth disease virus 2A sequence (F2A) to generate the pCR3.1‐OKT3‐FRB*‐F2A‐A3B1‐FKBP plasmid. A *Mlu*I restriction site was inserted upstream of the construct using two oligos from (Roche Diagnostics, Basel, Switzerland). The stop codon downstream OM‐OKT3‐FRB*‐His was removed by insertion of a synthetic sequence from GeneArt AG by *Not*I*/Xba*I digestion. Then, the whole construct was subcloned by *Mlu*I*/Xba*I digestion into a lentiviral pCCL plasmid vector upstream of a T2A‐green fluorescent protein (GFP) sequence [6], obtaining the pCCL‐OKT3‐FRB*‐F2A‐A3B1‐FKBP‐T2A‐GFP plasmid. The pCCL A3B1‐OKT3‐T2A‐tdTomato (tdTo) plasmid used as control in the experiments has been previously described [4]. A synthetic gene encoding the tags F/S followed by an anti‐EGFR V_HH_ [47, 48] and the FKPB sequence was synthesized by GeneArt AG. The synthetic DNA was then cloned into the bicistronic vector pCCL‐OKT3‐FRB*‐F2A‐A3B1‐FKBP‐T2A‐GFP using *Sph*I‐HF/*Xba*I restriction enzymes, obtaining the plasmid pCCL‐OKT3‐FRB*‐F2A‐EGFR‐FKBP‐T2A‐GFP. All restriction enzymes used were from New England BioLabs (Ipswich, MA, USA).

### 2.5 Cell Transfection

HEK293 or HEK293T cells were transfected with the appropriate expression plasmid (pCR3.1 or pCCL) using lipofectamine (Lipofectamine 3000 Reagent, Invitrogen, ThermoFisher Scientific) and were selected in DCM with 500 µg/mL geneticin (G‐418) and 150 µg/mL hygromycin B (Invitrogen) to generate stable cell lines. Non‐transfected cells (NTx) were used as negative controls. Supernatants from transiently and stably transfected cell populations were analyzed by Western blotting, ELISA, and flow cytometry.

### 2.6 Production of Lentiviral Particles

HEK293T cells were transfected with the corresponding pCCL plasmid, the packaging plasmids pMDLg/pRRE and pRSVrev, and envelope plasmid pMD2.G (all from PlasmidFactory GmbH, Bielefeld, Germany), using PEI (Polysciences, Warrington, PA, USA) as described [43]. Virus‐containing supernatants were collected, filtered and ultracentrifuged. The pellets were resuspended in RPMI‐1640, aliquoted and stored at ‒80°C. Viral titers were consistently ranged around 1 × 10^8^ transducing units/mL. Functional titers were determined by limiting dilution in Jurkat cells and analyzed for GFP or tdTo expression by flow cytometry.

### 2.7 Production of the CD3‐FRB* and CD19‐FKBP Purified Proteins

CD3‐FRB* and CD19‐FKBP were expressed in Expi293 cells (Thermo Fisher Scientific) via transient transfection using pCR3.1‐CD3‐FRB*‐Myc/His and pCR3.1‐F/S‐CD19‐FKBP plasmids, respectively, and ExpiFectamine 293 Reagent (Thermo Fisher Scientific), following the manufacturer's protocol. Transfected cells were cultured at 32°C in a humidified incubator with 5% CO_2_ on an orbital shaker platform and harvested after 6 days. The CD19‐FKBP protein was purified from the culture supernatant by Strep‐tag affinity chromatography using a StrepTrap XT column (Cytiva, Uppsala, Sweden) connected to an ÄKTA go system (Cytiva). The CD3‐FRB* protein was purified from the culture supernatant by immobilized‐metal affinity chromatography (IMAC) using a HisTrap Excel column (Cytiva) connected to an ÄKTA go system (Cytiva). Elutions were performed with 10 mM biotin for the Strep‐tag affinity or with 250 mM imidazole for IMAC. In both cases, the eluted fractions were concentrated using a Vivaspin 15R ultrafiltration device with a 10,000 MWCO (Sartorius, Göttingen, Germany). Concentrated proteins were further purified by size‐exclusion chromatography (SEC) on a Superdex 200 Increase 16/60 column (Cytiva) using an ÄKTA FPLC system (Cytiva), with phosphate‐buffered saline (PBS) as the running buffer at a flow rate of 0.1 mL/min. Protein purity was assessed by SDS‐PAGE and concentration was determined using a NanoDrop spectrophotometer (Thermo Fisher Scientific). Purified proteins were aliquoted, flash‐frozen in liquid nitrogen and stored at −80°C until use.

### 2.8 Analytical Determination of Rapalog Stability Over Time

The reversed phase high‐performance liquid chromatography (HPLC) with ultraviolet detection (UV) method for detection of rapalog (A/C heterodimerizer AP21967, Takara, San Jose, CA, USA) was first optimized, and a calibration curve was established using known concentrations of the compound in methanol and the quantification was done by UV. The compound was subsequently dissolved in the selected formulation (16.7% 1,2 propanediol, 22,5% PEG 400 and 1,25% Tween 80) at a final concentration of 0.6 mg/mL. The solution was stirred magnetically at 37°C for 1 h, followed by centrifugation for 1 h at room temperature. The stability of the formulated compound was evaluated over time, samples were diluted 1:100 with methanol at different times, analyzed, and quantified by integration of the chromatographic peak area. Compound concentrations were calculated using the calibration curve formula. The results indicate that the compound is chemically stable in the selected formulation for at least 72 h when stored at 4°C.

Chromatographic analysis was performed using reversed phase HPLC on a 3 µm C18 Phenomenex column (50 × 2 mm). The mobile phase consisted of solvent A (water containing 0.1% formic acid) and solvent B (acetonitrile containing 0.1% formic acid). A linear gradient from 95% A to 100% B was applied over 5 min. The compound was detected by UV spectroscopy and eluted with a retention time of 3.47 min.

### 2.9 Analytical Quantification of Rapalog in Plasma

Bioanalytical quantification of rapalog concentrations in plasma obtained from mice was performed by HPLC coupled with tandem mass spectrometry (MS; HPLC‐MS/MS). Mice received a single injection of rapalog or vehicle intraperitoneally (i.p.) at time 0. Peripheral blood was collected at 1 h and 24 h post‐injection and centrifuged to obtain plasma. Rapalog plasma concentrations were determined using a fully validated high HPLC–MS/MS method. Plasma samples were processed by protein precipitation with methanol containing an appropriate internal standard, followed by additional clean‐up via solid‐phase extraction prior to analysis. Chromatographic separation was achieved on an Agilent 1100 HPLC system equipped with a reversed‐phase C18 column (3 µm, Phenomenex, 50 × 2 mm), employing a gradient elution of water and methanol, both supplemented with 0.1% formic acid, under controlled flow conditions (600 µL/min) over 6 min. Detection was performed on a QTRAP5500 triple quadrupole mass spectrometer (Sciex, Framingham, MA, USA) operating in positive electrospray ionization mode, using multiple reaction monitoring for the selective detection of the analyte and internal standard. Rapalog plasma concentrations were determined by interpolation from a calibration curve previously constructed using plasma samples spiked with known concentrations of rapalog and processed under identical conditions.

### 2.10 T Cell Isolation

Peripheral blood mononuclear cells (PBMC) were isolated from peripheral blood of healthy volunteer donors (*n* = 15) by density gradient centrifugation using Lymphoprep (Axis‐Shield, Oslo, Norway). All donors provided written informed consent in accordance with the Declaration of Helsinki. CD3^+^ T cells were purified by negative selection using the human Pan T Cell Isolation Kit and LS columns (both from Miltenyi Biotec, Bergisch Gladbach, Germany) according to the manufacturer's instructions.

### 2.11 T Cell Transduction

Jurkat cells were transduced with the indicated lentiviruses at a multiplicity of infection (MOI) of 5 and expanded in RCM. PBMC were plate‐coated activated with 1 µg/mL anti‐CD3 (OKT3) and 1 µg/mL anti‐CD28 (CD28.2) monoclonal antibodies (mAb, BD Biosciences, San Jose, CA, USA) for 2 days and transduced (MOI of 10) with the indicated lentiviruses in the presence of 10 ng/mL interleukin (IL)‐7 and 10 ng/mL IL‐15 (Miltenyi Biotec, Bergisch Gladbach, Germany). T cells were expanded in RCM supplemented with IL‐7 and IL‐15 (10 ng/mL) for 5–10 days. Alternatively, after PBMC isolation, cells were subsequently activated and expanded for 24 h with Dynabeads Human T‐Activator CD3/CD28 (Gibco, ThermoFisher Scientific) at a cell‐to‐bead ratio of 1:3 in RCM at a concentration of 1 × 10^6^ cells/mL in the presence of 10 ng/ml IL‐7 and IL‐15. After 24 h, cells were transduced with lentiviruses encoding TCE^C^ or TCE^ON^ at a MOI of 5 and then cultured and expanded in RCM supplemented with IL‐7 and IL‐15 (10 ng/mL) for up to 14 days. Transduction efficiency in both Jurkat and primary T cells was determined by GFP or tdTo expression by flow cytometry. Non‐transduced Jurkat (J‐NT) or primary T cells (NT‐T) were used as negative controls.

### 2.12 Western Blotting

Supernatants from transfected HEK293 cells were separated under reducing conditions on 10%–20% Tris‐glycine gels (Life Technologies) and transferred to PVDF membranes (Merck Millipore). Then, membranes were blocked with PBS (Lonza) – 5% BSA (Sigma‐Aldrich) and probed with anti‐His (PentaHis clone, QIAGEN, Hilden, Germany) or anti‐FLAG (M2 clone, Sigma‐Aldrich) mAb, followed by some washes with PBS – 0.1% Tween20 (Sigma‐Aldrich) and incubation with a horseradish peroxidase (HRP)‐conjugated goat anti‐mouse IgG (Fc specific, Sigma‐Aldrich). Visualization of protein bands was performed with Pierce ECL Plus Western Blotting Substrate (ThermoFisher Scientific) and ChemiDoc MP Imaging System machine (Bio‐Rad Laboratories, Hercules, CA, USA). Analysis was performed using Image Lab software (Bio‐Rad).

### 2.13 Enzyme‐Linked Immunosorbent Assays and Cytokines Analysis

To study extracellular dimer formation, supernatants of stably transfected CD19‐FKBP or CD3‐FRB* HEK293 cells were mixed and treated or not with 500 nM of rapalog and incubated at 4 or 37°C for 24 h. Anti‐FLAG mAb (2.5 µg/mL in PBS, clone M2, Sigma‐Aldrich) was immobilized on Maxisorp plates (ThermoFisher Scientific) overnight at 4°C. After washing with PBS and blocking with PBS – 5% BSA, 100 µL of mixed supernatants were added and incubated for 1 h at room temperature. The wells were then washed with PBS‐0.05% (v/v) Tween‐20 and treated with HRP‐conjugated anti‐His mAb (Sigma‐Aldrich) diluted in PBS‐1% (w/v) BSA for 1 h. After washing, the plate was developed with tetramethylbenzidine (Sigma‐Aldrich) and read in a Multiskan FC Microplate Photometer (ThermoFisher Scientific). The results were analyzed and presented using Prism 7 (GraphPad Software, San Diego, CA). Cytokine secretion from in vitro supernatants (IFN‐γ, TNF‐α, IL‐2, IL‐6, GM‐CDF, IL1β, IL‐4, IL‐5, and IL‐10) was measured by multiplex bead‐based immunoassays using Cytokine Human Magnetic 10‐Plex Panel for Luminex Platform (ThermoFisher Scientific) or using a human IFN‐γ kit (Diaclone, Besancon Cedex, France), according to the manufacturer's instructions.

### 2.14 Flow Cytometry

The antibodies and reagents used for flow cytometry analysis are listed in Supplementary Table S1. Cell acquisition was performed on a FACSCanto II or BD LSRFortessa flow cytometer using BD FACSDiva software (BD Biosciences, San Jose, CA, USA) or on a DxFLEX flow cytometer using CytExpert software (Beckman Coulter, Brea, CA, USA). Analysis was performed using FlowJo V10 software (Tree Star, Ashland, OR, USA).

### 2.15 Binding Assays

The ability of the antibodies to bind to cell surface expressed CD19, EGFR or CD3, as well as dimers formation, was assessed by flow cytometry as previously described [48]. Briefly, CD19‐, EGFR‐ or CD3‐expressing or non‐expressing cells were incubated with supernatants from transiently or stably transfected HEK293 or HEK293T cells treated or not with 500 nM rapalog, followed by sample removal and incubation with a biotin‐labeled anti‐His mAb, an anti‐Strep mAb or with an Alexa Fluor 647‐conjugated anti‐G4S linker mAb. The cells were then washed, incubated with PE/APC‐conjugated streptavidin or PE/Alexa Fluor 647‐conjugated goat anti‐mouse IgG and washed before analysis and analyzed by flow cytometry.

### 2.16 T Cell Activation Assays

Non‐transduced or transduced Jurkat or primary CD3^+^ T cells were co‐cultured with tumor target cells at a 2:1 effector:target (E:T) ratio in the presence of supernatants from transfected HEK293T cells, with or without 500 nM rapalog. After 24 h, T cell activation was analyzed by flow cytometry using PE/PE‐Cy7 conjugated anti‐CD69 mAb. For ON/OFF kinetics experiments, non‐transduced Jurkat or J‐STAb‐T^ON^ cells were co‐cultured with SEM^WT^ target cells at a 2:1 effector‐to‐target ratio. Baseline CD69 expression was determined at day 0, followed by stimulation with 500 nM rapalog for 24 h. After the initial stimulation period, rapalog was either withdrawn or replenished daily, depending on the experimental condition. In withdrawal experiments, rapalog was removed after 24 h and cells were cultured in rapalog‐free medium for the remainder of the assay. In continuous stimulation experiments, 500 nM rapalog was added daily to maintain activation. For reactivation studies, rapalog was re‐administered on day 6 for 24 h following the withdrawal period. T cell activation was monitored longitudinally by flow cytometry through the analysis of CD69 expression using an APC‐conjugated anti‐CD69 monoclonal antibody.

### 2.17 Cytotoxicity Assays

Luciferase‐expressing CD19^+^, CD19^–^ EGFR^+^ or EGFR^–^ target cells were co‐cultured with non‐transduced (NT‐T) or transduced human CD3^+^ T cells at a 2:1 E:T ratio in the presence of supernatant from transfected HEK293T cells, with or without 500 nM rapalog. After 48 h, 20 µg/well D‐luciferin (Promega, Madison, WI, USA) was added before bioluminescence quantification using a Victor luminometer (PerkinElmer, Waltham, MA, USA) or CLARIOstar Plus microplate reader (BMG Labtech, Ortenberg, Germany). Percent specific cytotoxicity was calculated using the formula: 100 – [(bioluminescence of each sample*100) / mean bioluminescence of NT‐T cells]. A 100% lysis control was included by adding 5% (v/v) Triton X‐100 (Sigma‐Aldrich) to the target cells. Alternatively, CD19^+^ or CD19^−^ target cells were labeled with CellTrace Far Red or Violet (both from ThermoFisher Scientific) according to the manufacturer's instructions and then co‐cultured with transduced human CD3^+^ T cells at a 2:1 E:T ratio, with or without 500 nM rapalog. After 48 h, samples were analyzed by flow cytometry. Supernatants were collected and stored at –20°C for IFN‐γ secretion analysis.

### 2.18 Immunofluorescence and Confocal Microscopy

For immunological synapse (IS) studies, Raji cells labeled with 1 µM 7‐amino‐4‐chloromethylcoumarin (CellTracker Blue CMAC; Invitrogen) were incubated at a 1:1 E:T ratio with J‐NT, J‐CD19‐TCE^C^, or J‐CD19‐TCE^ON^ treated or not with rapalog at 37°C for 15 min. As an internal control, Raji cells were loaded with 1 µg/mL staphylococcal enterotoxin E (SEE) prior to incubation with J‐NT. Jurkat/Raji cell conjugates were then fixed with 4% paraformaldehyde (Sigma‐Aldrich) in PBS, permeabilized with 0.1% Triton X‐100 (Sigma‐Aldrich), and blocked with 10 µg/mL human gamma globulin. The samples were then stained with primary antibodies (Supplementary Table S1) for 1 h, washed with TBS (Tris 20 mM, NaCl 150 mM), and incubated with the appropriate secondary conjugated antibodies (Supplementary Table S1) and phalloidin (ThermoFisher Scientific) for 30 min. Finally, the coverslips were washed with TBS and distilled water before mounting with Mowiol medium (Sigma‐Aldrich). High‐resolution images of fixed specimens were captured using an SP‐8 confocal scanning laser microscope (Leica Microsystems, Wetzlar, Germany) with a 60X/1.35 oil immersion objective. CMAC, Alexa Fluor 488 and Alexa Fluor 594 secondary antibodies, and Alexa Fluor 647‐conjugated phalloidin were excited by 405, 488, 594, and 633 nm laser lines, respectively. Image analysis was performed using ImageJ freeware (National Institutes of Health, USA). Phospho‐tyrosine (pTyr) and F‐actin IS polarization were estimated using the synapse measure plugin of ImageJ. The percentage of activating interactions was obtained by visually counting the number of both activating and non‐activating interactions based on pTyr polarization. Results are expressed as mean ± standard deviation (SD).

### 2.19 In Vivo Studies

Rapalog was dissolved in vehicle solution (16.7% 1,2‐propanediol, 22.5% PEG‐400, 1.25% Tween‐80) to 0.6 mg/mL (3 mg/kg dose) prior to injection as previously described [25]. Briefly, rapalog was first dissolved in 100% 1,2‐propanediol by vigorous vortexing or sonication and then diluted to final working concentration with an appropriate mixture of PEG‐400, Tween‐80 and water. Working stocks of rapalog or vehicle were stored at 4°C between injections. For the short‐term in vivo experiment, 8‐12‐week‐old female NSG mice were injected i.p. with 5 × 10^6^ NALM6 cells. After 24 h, 5 × 10^6^ transduced human T cells were injected i.p., followed by i.p. injection of rapalog (3 mg/kg) or vehicle (*n* = 3 mice per group) on the opposite flank. Three additional injections of rapalog or vehicle were made at 8, 24, and 32 h after the first injection. Mice were euthanized 72 h after the start of the experiment, peritoneal lavage was collected as described [25], and samples were analyzed by flow cytometry. For the pharmacokinetic analysis of rapalog in vivo, 8–12‐week‐old female NXG mice received a single injection of rapalog i.p. (3 mg/kg) or vehicle (*n* = 3 mice per group). Peripheral blood was collected 1 and 24 h after injection and centrifuged to obtain plasma. Rapalog plasma concentrations were determined as described above by HPLC‐MS/MS. Each plasma sample was analyzed in technical triplicate. For the medium‐term in vivo experiment, 8–12‐week‐old female NXG mice (*n* = 5 mice per group) were injected subcutaneously (s.c.) with 0.5 × 10^6^ NALM6 cells together with 2.5 × 10^6^ non‐transduced (NT‐T), STAb‐T^C^, or STAb‐T^ON^ primary T cells resuspended in 80% Corning Matrigel Basement Membrane Matrix (Corning, NY, USA). One week later (day 7), mice received a second intravenous (i.v.) infusion of 2.5 × 10^6^ T cells; following each T‐cell infusion, mice received two daily doses of rapalog (3 mg/kg) or vehicle during the subsequent two days. In the rapalog withdrawal group, administration was discontinued on day 10, whereas all other groups continued to receive rapalog or vehicle once daily until day 19. Tumor growth was monitored twice weekly by caliper measurements, and body weight was recorded throughout the study. Tumor volumes were calculated using the formula V = (D × d^2^)/2 mm^3^, where *D* corresponds to the largest tumor diameter and *d* to the shortest diameter. Peripheral blood was collected from mice at the study endpoint. An aliquot was analyzed by flow cytometry to assess T‐cell persistence. Human cells were identified by HLA‐ABC expression, followed by gating on CD2^+^CD45^+^ T cells. T‐cell persistence was expressed as the percentage of CD2^+^CD45^+^ T cells within the HLA‐ABC^+^ cell population. The remaining blood was centrifuged to obtain plasma for the assessment of circulating TCE activity. Plasma samples were evaluated using a functional Jurkat activation assay in which Jurkat cells were co‐cultured with CD19^+^ NALM6 target cells. T‐cell activation was quantified by measuring CD69 expression (median fluorescence intensity, MFI; CD45^+^ gate) by flow cytometry. In parallel, a blinatumomab standard curve was generated under identical assay conditions, and blinatumomab‐equivalent activity in plasma samples was determined by interpolation from the standard curve.

### 2.20 Statistical Analysis

Statistical tests indicated in the figure legends were performed using Prism 6/8 (GraphPad Software, La Jolla, CA, USA). Experimental results are expressed as mean ± standard deviation (SD) or mean ± standard error of the mean (SEM). Significance was considered only when p‐values were less than 0.05 (*p < 0.05; **p < 0.01; ***p < 0.001, ****p < 0.0001).

## 3 Results

### 3.1 Engineering and Validation of the TCE^ON^ Platform

The conventional anti‐CD19 TCE (TCE^C^) consists of two scFvs specific for CD19 and CD3 linked by a short peptide linker [4] (Figure S1a,b). To generate a drug‐regulated ON‐switch variant (TCE^ON^), we fused the anti‐CD19 and anti‐CD3 scFvs to heterodimerizing domains: FK506‐binding protein (FKBP) and the T2089L mutant of the FKBP–rapamycin‐binding domain (FRB*), respectively. The CD19‐FKBP construct was tagged with FLAG and StrepII epitopes (Figure S1c,d), while the CD3‐FRB* construct contained a poly‐histidine tag (Figure S1e,f). In the presence of rapamycin or its analog AP21967 (rapalog), FKBP and FRB* heterodimerize, enabling the assembly of the CD19‐FKBP and CD3‐FRB* modules into a functional anti‐CD19 × anti‐CD3 TCE‐like structure, referred to as TCE^ON^ (Figure S1g). HEK293 cells were transfected with each construct, and western blot analysis confirmed efficient secretion of CD19‐FKBP and CD3‐FRB* proteins (Figure S2a), with migration patterns consistent with their predicted molecular weights (42.4 and 41.3 kDa, respectively). The functionality of both modules was validated in a cellular context by flow cytometry: CD19‐FKBP bound selectively to CD19^+^ Raji cells, while CD3‐FRB* recognized CD3^+^ Jurkat T cells; neither construct bound CD19^−^CD3^−^ K562 cells (Figure S2b). Rapalog‐dependent heterodimerization between CD19‐FKBP and CD3‐FRB* was demonstrated using a sandwich ELISA based on differential epitope tagging. Briefly, conditioned media (CM) from transfected HEK293 cells were either mixed or kept separate, incubated overnight at 37 °C in the presence or absence of rapalog, and added to anti‐FLAG–coated plates. Bound complexes were detected using an anti‐His mAb. A signal was observed exclusively for the CD19‐FKBP:CD3‐FRB* mixture in the presence of rapalog, indicating successful assembly of a bispecific heterodimer (Figure S2c). To assess dose‐dependent binding capacity, CM from separately transfected cells were combined and incubated with increasing concentrations of rapalog. ELISA revealed a proportional increase in signal intensity with higher rapalog concentrations, confirming a tunable, drug‐dependent assembly of the TCE^ON^ complex (Figure S2d). Based on these data, 500 nM rapalog was selected for all subsequent in vitro studies, as this concentration consistently produced near‐maximal assembly of the TCE^ON^ complex.

### 3.2 Inducible Assembly of Secreted Split TCE Modules Into Functional TCE Constructs

HEK293 cells were transfected either individually with CD19‐FKBP or CD3‐FRB* constructs, or simultaneously with both (referred to as 2Tx cells), and selected using geneticin and/or hygromycin. CM from 2Tx cells bound to CD19^+^ Raji cells when detected with an anti‐StrepII mAb (recognizing CD19‐FKBP), and to CD3^+^ Jurkat T cells when detected with an anti‐His mAb (recognizing CD3‐FRB*), confirmed secretion of both molecules in a functionally active form (Figure S2e). Neither mono‐ nor double‐transfected CM showed binding to CD19^−^CD3^−^ K562 cells, and CM from non‐transfected (NTx) cells did not bind any of the tested cell lines (Figure S2e). We next investigated whether CD19‐FKBP and CD3‐FRB* could heterodimerize to form a functional TCE‐like molecule in a rapalog‐dependent manner, taking advantage of their distinct epitope tags (Figure S1). Since CD3‐FRB* lacks a StrepII tag, binding of CM to Jurkat T cells detected with an anti‐StrepII antibody would indicate dimerization with CD19‐FKBP. Conversely, CD19‐FKBP lacks a His tag, so detection on Raji cells with an anti‐His antibody would similarly require rapalog‐induced heterodimer formation (Figure S1). Stable transfectants were cultured in the presence or absence of rapalog for 24 h, and CM binding was analyzed by flow cytometry. As expected, in the absence of rapalog, CM from single‐ or double‐transfected cells did not bind Jurkat T cells when probed with anti‐StrepII mAb (Figure S2f, top). However, CM from 2Tx cells cultured with rapalog exhibited clear binding, indicating formation of the bispecific complex. Similarly, anti‐His detection revealed that only rapalog‐treated 2Tx CM bound Raji cells, confirming dimerization with CD19‐FKBP (Figure S2f, bottom). To assess whether the CD19‐FKBP/CD3‐FRB* heterodimer formed in the presence of rapalog possesses functional TCE activity, primary T cells were co‐cultured at a 2:1 effector‐to‐target (E:T) ratio with either CD19^−^ K562 or CD19^+^ Raji cells in the presence of CM from NTx, single‐transfected, or 2Tx HEK293 cells, with or without rapalog. T cell activation was evaluated by measuring CD69 surface expression by flow cytometry (Figure S2g). CD69 upregulation was observed exclusively in co‐cultures with Raji cells and CM from 2Tx cells treated with rapalog (Figure S2g, bottom), reaching levels comparable to those induced by the conventional CD19‐TCE (Figure S2g, top). No CD69 expression was detected under any other condition, including CM from 2Tx cells without rapalog or CM from single‐transfected or NTx cells, regardless of rapalog treatment. These results confirm that the rapalog‐induced heterodimer functions as a bona fide bispecific TCE capable of redirecting T cell activation in an antigen‐specific manner.

### 3.3 Functional Characterization of Purified CD3‐FRB* and CD19‐FKBP Modules

To validate the modular design, CD3‐FRB* and CD19‐FKBP proteins were individually expressed and purified. The CD19‐FKBP module was purified by Strep‐tag affinity chromatography method, whereas the CD3‐FRB* module was purified using IMAC. Under reducing SDS–PAGE, both modules migrated as single bands corresponding to their expected monomeric sizes (≈41–42 kDa; Figure S3a). Binding specificity was examined by flow cytometry across a range of protein concentrations. CD19‐FKBP bound selectively to SEM^WT^ cells at 2 nM, with no detectable interaction with SEM^KO^, whereas CD3‐FRB* recognized CD3^+^ Jurkat T cells at 20 nM, confirming antigen‐specific recognition of each module (Figure S3b). Functional activation was assessed by co‐culturing Jurkat cells with SEM^WT^ or SEM^CD19KO^ cells in the presence of increasing concentrations of both proteins and rapalog. CD69 upregulation occurred only in CD19^+^ co‐cultures and in the presence of 500 nM rapalog, increasing from robust activation at 1 nM to maximal activation at 10 nM (Figure S3c). Finally, primary T cells were co‐cultured with SEM^WT^ or SEM^CD19KO^ target cells at a 2:1 E:T ratio for 48 h in the presence of increasing concentrations (0.1–1 nm) of CD3‐FRB* and CD19‐FKBP protein modules. Luminescence‐based assays revealed dose‐dependent cytotoxicity, reaching ∼80% specific killing in the presence of rapalog, whereas negligible cytotoxicity was observed in its absence or against SEM^CD19KO^ targets (Figure S3d).

### 3.4 Coordinated Expression of TCE^ON^ Components Using a 2A Peptide‐Based System

To co‐express the CD19‐FKBP and CD3‐FRB* modules (TCE^ON^), we generated a bicistronic construct using a 2A peptide (Figure S1h). Schematic representation in Figure 1a shows how antigen recognition (binding) and activation occurs in the presence of rapalog. HEK293 cells were transfected with each construct and the resulting CM were used to assess antigen binding and T cell activation. CM from NTx, CD19‐FKBP, CD3‐FRB*, and TCE^ON^ transfections were incubated with K562 or Raji cells. Flow cytometry confirmed that both components were efficiently secreted and retained antigen specificity: CD19‐FKBP and TCE^ON^ bound selectively to CD19^+^ Raji cells, whereas CD3‐FRB* and TCE^ON^ recognized CD3 on Jurkat T cells, with no detectable binding to antigen‐negative K562 cells (Figure 1b). To evaluate functional T cell activation, CM from NTx, TCE^C^ and TCE^ON^ cultures were added to Jurkat cells co‐cultured with CD19^−^ K562 or CD19^+^ NALM6 targets at a 2:1 E:T ratio for 24 h in the presence or absence of 500 nM rapalog. As expected, TCE^C^ induced CD69 upregulation upon co‐culture with NALM6 cells in a rapalog‐independent manner. In contrast, TCE^ON^ induced CD69 upregulation only in NALM6 co‐cultures and exclusively in the presence of rapalog, whereas no activation was detected under any condition with antigen‐negative K562 cells (Figure 1c). To assess cytotoxic activity, primary human T cells were co‐cultured with luciferase‐expressing CD19^−^ or CD19^+^ target cells at a 2:1 E:T ratio, in the presence of CM from transfected HEK293 cells, with or without 500 nM rapalog, for 48 h. CM from TCE^ON^ cells induced potent and specific lysis of CD19^+^ targets only in the presence of rapalog, reaching levels comparable to those achieved with the conventional TCE^C^ (Figure 1d). No cytotoxicity was observed in the absence of rapalog or against CD19^−^ target cells.

**FIGURE 1:**
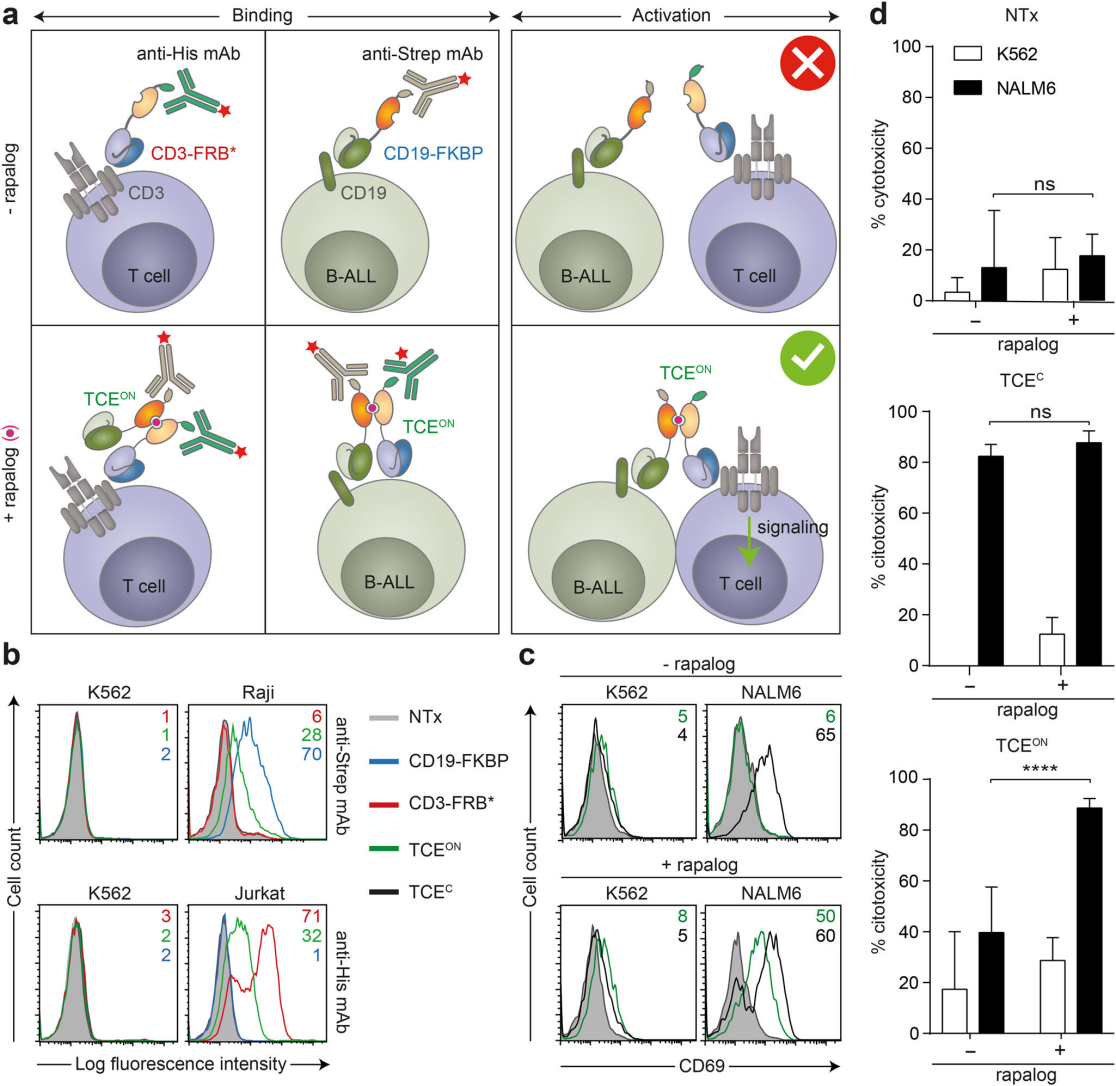
Characterization of the 2A‐based CD19‐TCE^ON^. (a) Schematic representation of the cell binding (left) and activation (right) depending on the presence of rapalog in the CM before and after heterodimerization. (b) Binding detection after antigen recognition with anti‐Strep mAb and anti‐His mAb of the modules of the TCE^ON^ (CD19‐FKBP and CD3‐FRB*) in CM from HEK293 cells transfected with the 2A‐based construct of the TCE^ON^. (c) CD69 expression in primary T cells after co‐culture with K562 or NALM6 cells in presence of CM from HEK293 cells transfected with the 2A‐based construct of the TCE^ON^ or TCE^C^ treated or not with rapalog. (d) Specific cytotoxicity analysis after 48‐h co‐culture of primary T cells with K562^Luc^ or NALM6^Luc^ in presence of CM from NTx, TCE^C^‐ or TCE^ON^‐transfected HEK293 cells, treated or not with rapalog. Data represent mean ± SEM (*n* = 3) and statistical significance was calculated by two‐way ANOVA test corrected by Tukey's multiple comparisons test; ****p < 0.0001; ns, non‐significant. CM, conditioned media; NTx, non‐transfected.

### 3.5 Generation and Characterization of STAb‐T^ON^ Cells

The TCE^C^ and TCE^ON^ constructs were cloned under the control of the EF1α promoter into T2A‐based bicistronic lentiviral vectors encoding the fluorescent reporter proteins tdTo and GFP, respectively (Figure S4a,b). Lentiviral vectors were used to transduce Jurkat T cells (Figure 2a), generating the J‐STAb‐T^C^ and J‐STAb‐T^ON^ cell lines. To determine whether the activation of J‐STAb‐T^ON^ cells could be modulated by rapalog concentration, J‐NT, J‐STAb‐T^C^, and J‐STAb‐T^ON^ cells were co‐cultured with NALM6 target cells in the presence of increasing rapalog concentrations of rapalog (1–500 nM). As expected, J‐NT cells remained unresponsive while J‐STAb‐T^C^ cells were robustly activated by NALM6 cells irrespective of rapalog concentration. In contrast, J‐STAb‐T^ON^ activation increased progressively with increasing rapalog concentrations, as reflected by the proportion of CD69^+^ cells (Figure 2b). To investigate the ON/OFF kinetics of the TCE^ON^ platform, J‐STAb‐T^ON^ cells were cultured with SEM^WT^ target cells and exposed to rapalog for 24 h. Rapalog withdrawal resulted in a progressive decline in CD69 expression, which gradually returned to baseline, whereas continuous rapalog exposure maintained sustained T cell activation throughout the experiment (Figure 2c). Importantly, re‐exposure of rapalog on day 6 fully restored CD69 expression, demonstrating that T cell activation can be dynamically regulated through repeated cycles of rapalog withdrawal and re‐exposure.

**FIGURE 2:**
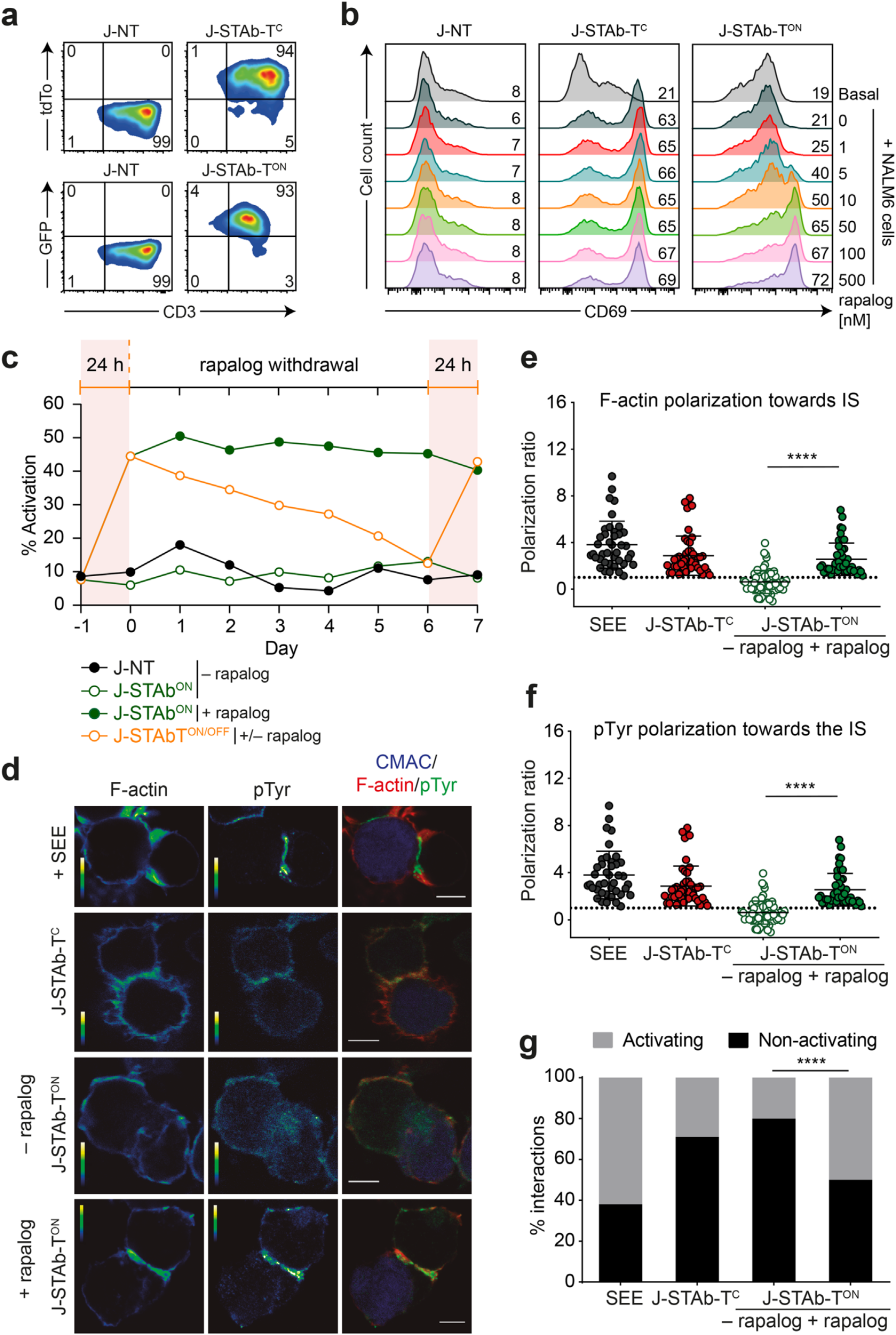
Characterization of Jurkat STAb‐T^ON^ cells. (a) Percentage of reporter‐positive cells in J‐STAb^C^ (tdTo) and J‐STAb^ON^ (GFP) populations. (b) CD69 expression by J‐NT, J‐STAb^C^, and J‐STAb^ON^ cells after co‐culture with NALM6 cells in the presence of increasing concentrations of rapalog (0‐500 nM). (c) ON/OFF kinetics of rapalog‐dependent CD69 activation. J‐NT, J‐STAb^C^ and J‐STAb^ON^ cells were co‐cultured with with SEM^WT^ cells at a 2:1 E:T ratio. Basal CD69 expression was measured at day 0, followed by the addition of 500 nM rapalog for 24 h. Rapalog was then withdrawn from the J‐STAb^ON^ cells (J‐STAb^ON/OFF^ group), whereas J‐STAb^ON^ (+ rapalog) group received daily rapalog re‐dosing throughout the experiment. On day 6, rapalog was re‐administered to the J‐STAb^ON/OFF^ group for 24 h, and CD69 expression was analyzed on day 7. (d) J‐NT, J‐STAb^C^ and J‐STAb^ON^ IS formation in the presence or absence of rapalog with Raji cells assessed by confocal microscopy. pTyr and F‐actin (pseudocolor) distribution and IS polarization are displayed (left and middle columns). Calibration bar of the pseudocolor is indicated. Merged images (right column) show pTyr (green), F‐actin (red) and CMAC (blue, Raji cells). Scale bars correspond to 5 µm. (e‐f) F‐actin (e) and pTyr (f) polarization to the IS. Each dot represents the value of individual cell interactions obtained from *n* = 2 independent experiments. The dashed line indicates the polarization ratio equals 1, meaning there is no polarization. (g) Percentage of activating interactions in each co‐culture. Results from *n* = 2 experiments are shown. Data in (e, f) represent mean ± SD and statistical significance was calculated by one‐way ANOVA test corrected by Tukey's multiple comparisons test (e, f) or by Fisher's exact tests (g); ****p < 0.0001. NT, non‐transduced.

Immunological synapse (IS) formation between J‐STAb‐T^C^ or J‐STAb‐T^ON^ cells and CD19^+^ Raji cells was analyzed by confocal microscopy. Synapse architecture and early TCR signaling were assessed by staining for F‐actin and phospho‐tyrosine (pTyr), respectively (Figure 2d). Both J‐STAb‐T^C^ and J‐STAb‐T^ON^ cells (in the presence of rapalog) exhibited comparable recruitment of F‐actin and pTyr to the IS, whereas J‐STAb‐T^ON^ cells without rapalog did not (Figure 2e,f). Interestingly, the proportion of activating interactions was higher in J‐STAb‐T^ON^ cells in the presence of rapalog than in J‐STAb‐T^C^ cells (Figure 2g), although both platforms induced comparable levels of F‐actin polymerization and phosphotyrosine (pTyr) accumulation. Co‐cultures of J‐NT cells with SEE‐loaded Raji cells served as a positive control.

Primary T cells were transduced with lentiviral vectors encoding either TCE^C^ or TCE^ON^ (Figure 3a), generating STAb‐T cells secreting TCE^C^ (STAb‐T^C^) or STAb‐T cells secreting TCE^ON^ (STAb‐T^ON^), respectively. Comparable transduction efficiencies ranging from 41% to 81% were obtained (Figure 3b). The proportion of CD4^+^ and CD8^+^ cells (Figure 3c) and the relative distribution of naïve (T_N_), central memory (T_CM_), effector memory (T_EMRA_) and effector (T_EF_) T cell subsets (Figure 3d) were similar in non‐transduced (NT‐T), STAb‐T^C^ and STAb‐T^ON^ cells. To estimate the relative abundance of the two TCE^ON^ modules in conditioned media, purified CD3‐FRB* and CD19‐FKBP proteins were first used to generate standard curves, enabling independent quantification of CD3‐FRB* and CD19‐FKBP concentrations (Figure S5a). CM collected from transiently transfected HEK293T cells expressing the TCE^ON^ construct (HEK293T^ON^), as well as from transduced Jurkat cells (J‐STAb^ON^) and primary T cells (STAb‐T^ON^), were then analyzed using the same binding‐based assay. Across all three production platforms, the estimated concentrations of CD3‐FRB* and CD19‐FKBP were highly comparable, yielding CD3‐FRB*/CD19‐FKBP ratios close to unity (0.999, 1.236 and 1.036, respectively), with an overall mean ratio of 1.09 (Figure S5b). These findings indicate that the two TCE^ON^ modules are secreted at near‐equimolar levels regardless of the cellular production platform.

**FIGURE 3:**
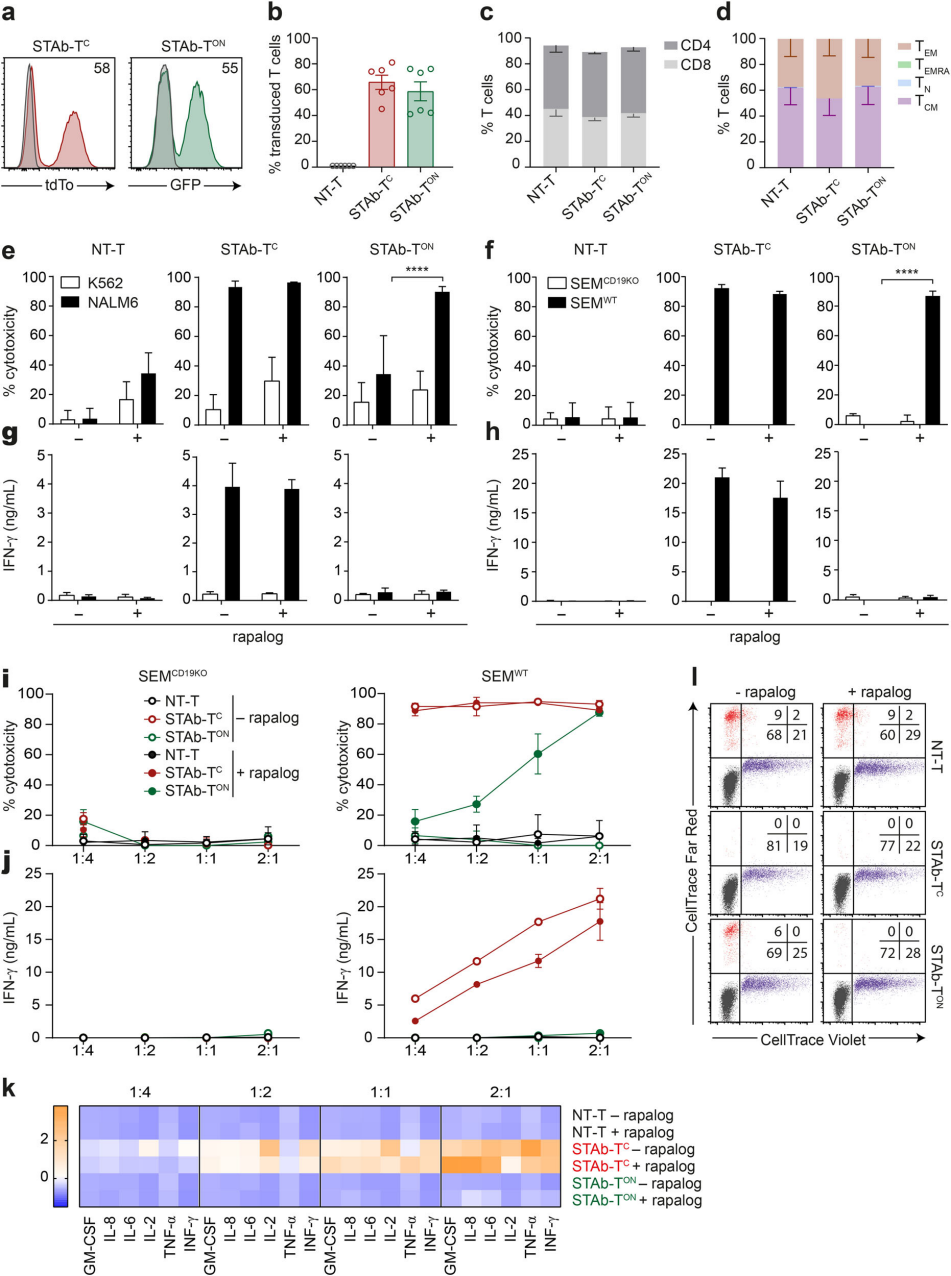
Rapalog‐dependent antitumor activity of inducible primary STAb‐T^ON^ cells in vitro. (a) Percentage of reporter gene expression in primary T cells transduced with TCE^C^ (tdTo) and TCE^ON^ (GFP). One representative transduction is shown. (b) GFP and tdTo expression in STAb‐T^ON^ and STAb‐T^C^ cells. (c) Proportion of CD4^+^ and CD8^+^ T cells. (d) Naïve (T_N_), central memory (T_CM_), effector memory (T_EMRA_) and effector (T_EF_) lymphocytes. Data in (c) and (d) represent mean ±standard deviation (SEM) of four independent experiments (*n* = 3). (e and f) Specific cytotoxicity analyzed by luminescence after 48 h co‐culture of NT‐T or transduced primary T cells with K562^Luc^ or NALM6^Luc^ (e) and SEM^WTLuc^ or SEM^KOLuc^ (f) at 2:1 E:T ratio in presence or absence of rapalog. Data represent mean ± SEM from *n* = 3 independent experiments performed by triplicate, and statistical significance was calculated by one‐way ANOVA test corrected by Tukey's multiple comparisons test; ****p < 0.0001. (g anf h) IFN‐γ secretion analyzed by luminescence after 48 h co‐culture of NT‐T or transduced primary T cells with K562^Luc^ or NALM6^Luc^ (g) and SEM^WTLuc^ or SEM^KOLuc^ (h) at 2:1 E:T ratio in presence or absence of rapalog. Data represent mean ± SEM from *n* = 3 independent experiments performed by triplicate, and statistical significance was calculated by one‐way ANOVA test corrected by Tukey's multiple comparisons test; ****p < 0.0001. (i and j) Specific cytotoxicity (i) and IFN‐γ secretion (j) assessed using NT‐T primary T cells (black), STAb‐T^C^‐transduced cells (red) and STAb‐T^ON^‐transduced cells (green) with or without rapalog after 48‐h co‐culture with SEM^KOLuc^ (left) or SEM^WTLuc^ (right) targets at indicated E:T ratios. Data represent mean ± SEM triplicates. (k) Pro‐inflammatory cytokine secretion (GM‐CSF, IL‐8, IL‐6, IL‐2, TNF‐α, IFN‐γ) quantified by multiplex bead‐based immunoassay in supernatants collected from the cytotoxicity assay shown in (i) at the indicated E:T ratios. (l) Specific cytotoxicity analyzed by flow cytometry mediated by NT‐T or transduced primary T cells after 72‐h co‐culture with previously labeled K562 and NALM6 cells (CellTrace Violet and CellTrace Far Red, respectively).

NT‐T, STAb‐T^C^ and STAb‐T^ON^ cells were subsequently co‐cultured with K562 or NALM6 cells (Figure 3e), or with SEM^WT^ or SEM^CD19KO^ cells (Figure 3f), at a 2:1 E:T ratio in the presence or absence of rapalog. After 48 h, target cell killing was assessed by luminescence, and culture supernatants were analyzed for IFN‐γ secretion. STAb‐T^ON^ cells mediated specific cytotoxicity against NALM6 and SEM^WT^ cells only in the presence of rapalog, whereas STAb‐T^C^ cells induced target cell lysis independently of rapalog (Figure 3e,f). Notably, STAb‐T^ON^ cells did not produce increased levels of IFN‐γ under any of the conditions tested, whereas STAb‐T^C^ cells secreted substantial amounts of IFN‐γ following co‐culture with NALM6 and SEM^WT^ cells, irrespective of the presence of rapalog (Figure 3g,h).

Furthermore, STAb‐T^C^ or STAb‐T^ON^ cells were co‐cultured with either SEM^WT^ or SEM^CD19KO^ target cells across a range of E:T ratios. In co‐cultures with SEM target cells, STAb‐T^C^ cells induced robust cytotoxicity (≥80%) across all E:T ratios, independently of rapalog. In contrast, STAb‐T^ON^ cells exhibited cytotoxic activity in the presence of rapalog, with maximal killing at a 2:1 ratio and markedly reduced activity at lower ratios (Figure 3i). Co‐cultures of STAb‐T^C^ cells with SEM targets exhibited an effector cell dose‐dependent increase in IFN‐γ secretion that was unaffected by rapalog treatment, whereas STAb‐T^ON^:SEM co‐cultures produced no detectable IFN‐γ under any condition. Neither STAb‐T^C^ nor STAb‐T^ON^ cells elicited detectable cytotoxicity or IFN‐γ secretion following co‐culture with SEM^CD19KO^ cells (Figure 3j). To further characterize the functional profile of TCE^ON^‐mediated T cell activation, cytokine secretion was quantified in supernatants collected from cytotoxicity assays using a multiplex Luminex panel (Figure 3k). Consistent with the reduced IFN‐γ production, STAb‐T^ON^ cells secreted significantly lower levels of multiple pro‐inflammatory cytokines, including TNF‐α, IL‐2, IL‐6, and GM‐CSF, than conventional STAb‐T^C^ cells. IL‐1β remained undetectable in all engineered and NT‐T groups. Likewise, the Th2/regulatory cytokines IL‐4, IL‐5, and IL‐10 were not detected under any experimental condition (Figure S6). Together, these findings indicate that TCE^ON^ activation is associated with a broadly attenuated inflammatory cytokine response while preserving antigen‐specific cytotoxic activity.

To further evaluate target‐specific cytotoxic, CellTrace Violet‐labeled K562 and CellTrace Far Red‐labeled NALM6 cells were mixed and co‐cultured with NT‐T, STAb‐T^C^, or STAb‐T^ON^ cells in the presence or absence of rapalog. STAb‐T^C^ cells selectively eliminated NALM6 cells irrespective of rapalog treatment, whereas STAb‐T^ON^ cells mediated NALM6‐specific killing only in the presence of rapalog (Figure 3l).

### 3.6 Inducible STAb‐T^ON^ T Cells Mediate Rapalog‐Dependent Anti‐Tumor Activity in Vivo

To evaluate the anti‐tumor activity of STAb‐T^ON^ cells in vivo, a short‐term study was conducted in which NSG mice were injected intraperitoneally (i.p.) with NALM6 cells (Figure S7a). Twenty‐four hours later, mice received an i.p. injection of STAb‐T^C^ or STAb‐T^ON^ cells together with the first dose of rapalog or vehicle. Three additional doses of rapalog or vehicle were administered at 8, 24, and 32 h after the initial treatment (Figure S7b). After 48 h, mice were euthanized and peritoneal lavage samples were analyzed by flow cytometry. A readily detectable population of human CD19^+^ cells persisted in mice treated with rapalog alone, whereas no residual NALM6 cells were detected in mice receiving STAb‐T^C^ cells. A limited reduction in NALM6 cell burden was observed in mice treated with STAb‐T^ON^ cells plus vehicle, suggesting minimal basal activity. In contrast 2 out of 3 mice treated with STAb‐T^ON^ cells in together with rapalog showed complete elimination of NALM6 cells (Figure S7b), demonstrating that STAb‐T^ON^ cells remain functional in vivo and mediate potent rapalog‐dependent anti‐tumor activity. To support the in vivo dosing regimen, we first assessed the stability of rapalog under the experimental conditions. Rapalog remained stable for at least 3–4 days after reconstitution (Figure S7c), indicating that its physicochemical integrity was maintained throughout the the treatment period. To characterize its in vivo pharmacokinetic, NXG mice received a single i.p. injection of rapalog, and plasma concentrations were quantified by HPLC‐MS/MS at 1 and 24 h post‐administration (Figure S7d). Rapalog reached readily detectable plasma concentrations at 1 h but declined below the lower limit of quantification (LLOQ) in most animals by 24 h, consistent with rapid systemic clearance (Figure S7e).

To further evaluate the in vivo activity of the TCE^ON^ system, we performed a medium‐term xenograft study using an expanded cohort of NXG mice (*n* = 5 per group), an optimized rapalog dosing regimen, and an additional STAb‐T^ON^ group in which rapalog treatment was discontinued on day 10 (Figure 4a). Both STAb‐T^C^ and STAb‐T^ON^ cells significantly delayed tumor growth compared with NT‐T cells (Figure 4b). As expected, STAb‐T^C^ cells achieved the greatest antitumor efficacy; however, continuous rapalog administration also enabled robust tumor control in mice treated with STAb‐T^ON^ cells. In contrast, rapalog withdrawal on day 10 led to accelerated tumor progression compared to continuous treatment, demonstrating that sustained antitumor activity activity depends on continued dimerizer administration. No significant differences in body weight were observed among the experimental groups throughout the study, indicating that all treatments were well tolerated (Figure 4c). Analysis of peripheral blood at the study endpoint revealed reduced persistence of NT‐T cells, whereas higher numbers of circulating human T cells were detected in all STAb‐T‐treated groups (Figure 4d). However, functional analysis of plasma samples detected no measurable circulating TCE activity, with all samples remaining below the lower limit of quantification (Figure 4e), indicating minimal systemic exposure to the secreted TCE despite prolonged persistence of STAb‐T cells.

**FIGURE 4:**
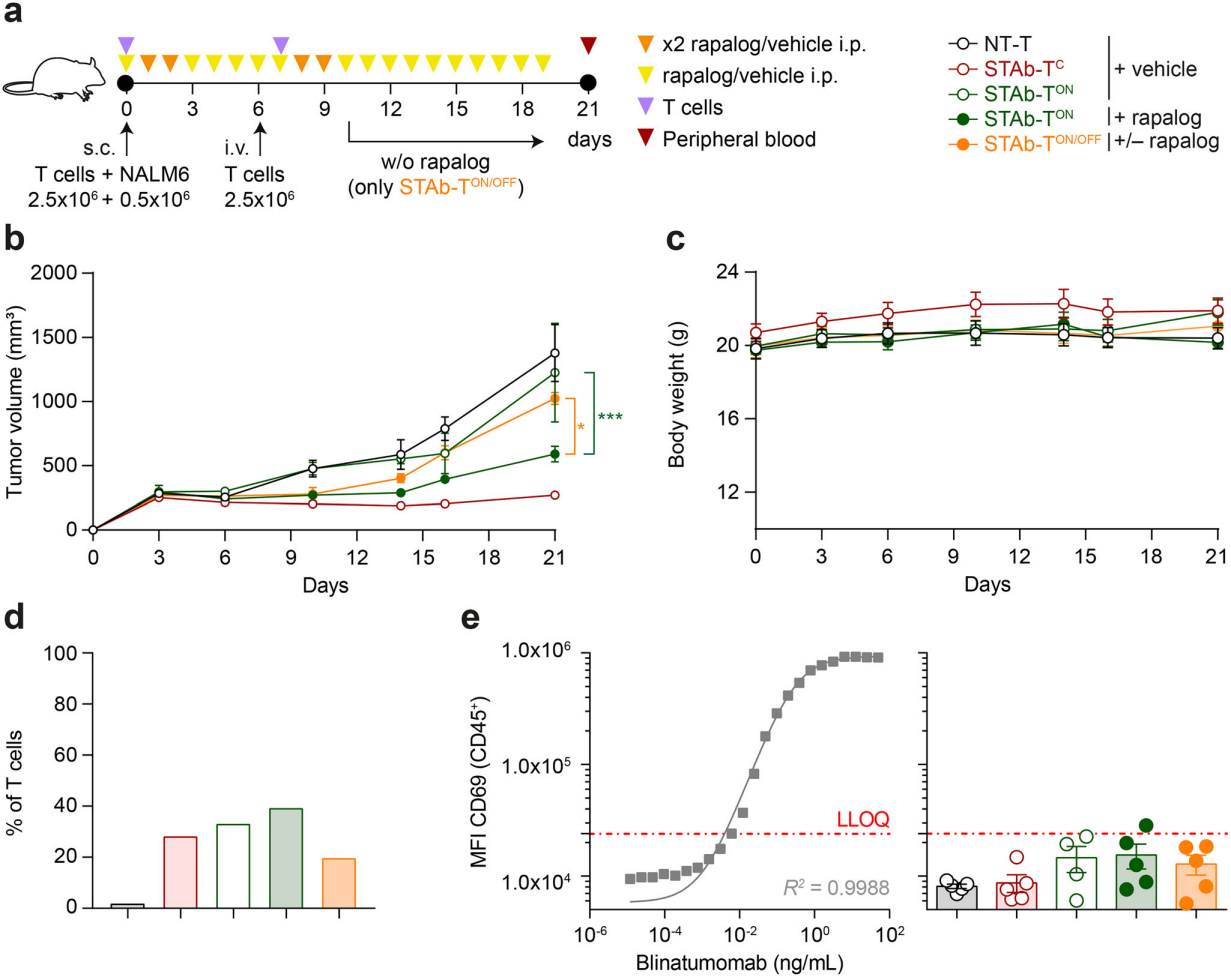
Rapalog‐dependent antitumor activity of inducible STAb‐T^ON^ cells in vivo. (a) Medium‐term in vivo study. NXG mice (*n* = 5 per group) were subcutaneously (s.c.) inoculated with 0.5 × 10^6^ NALM6 cells together with 2.5 × 10^6^ NT‐T, STAb‐T^C^, or STAb‐T^ON^ cells. A second intravenous (i.v.) infusion of 2.5 × 10^6^ T cells was administered on day 7. Rapalog was administered daily by intraperitoneal (i.p.) injection throughout the study except in the STAb‐T^ON^ ± rapalog group, in which dosing ceased on day 10. (b) Tumor volume measurements. (c) Body weight. Data represent mean ± SEM and statistical significance was determined by two‐way ANOVA with Tukey's multiple comparisons test; *p < 0.05; ***p < 0.001. NT, non‐transduced. (d) T cell persistence at the study endpoint. Peripheral blood samples from mice within each experimental group (*n* = 5) were pooled prior to flow cytometric analysis. Human cells were identified by HLA‐ABC expression, followed by gating on CD2^+^CD45^+^ T cells. The graph shows the percentage of human CD2^+^CD45^+^ T cells within the HLA‐ABC^+^ population. Each bar represents one pooled sample per experimental group. (e) Assessment of circulating TCE activity at the study endpoint using a Jurkat T cell activation assay. Left, blinatumomab standard curve generated by measuring CD69 expression (MFI, CD45^+^ gate) in Jurkat cells co‐cultured with CD19^+^ NALM6 target cells. The standard curve was fitted by nonlinear regression (four‐parameter logistic model; R^2^ = 0.9988) and used to estimate blinatumomab‐equivalent TCE activity in plasma samples. The red dashed line indicates the lower limit of quantification (LLOQ). Right, blinatumomab‐equivalent TCE activity in plasma samples from the indicated experimental groups. Each dot represents one mouse; bars indicate mean ± SEM. All samples were below the LLOQ, precluding reliable quantification of circulating TCE activity.

### 3.7 Extension of the TCE^ON^ Platform to an EGFR‐Targeting Solid Tumor Model

To investigate whether the TCE^ON^ strategy could be applied to a different tumour antigen, we generated an EGFR‐targeting TCE^ON^ construct by replacing the CD19‐binding scFv with an anti‐EGFR V_HH_ while preserving the rapalog‐inducible FKBP/FRB* assembly mechanism (Figure S8 and Figure 5a). Secretion of the individual modules into CM from transfected HEK293T cells and their rapalog‐dependent assembly upon antigen binding were confirmed by ELISA using an anti‐G4S linker mAb (Figure 5b). We next evaluated the functionality of the EGFR‐targeting TCE^ON^ construct in co‐cultures of primary T cells with EGFR‐positive tumor cell lines. CM from HEK293T cells expressing the EGFR‐targeting TCE^ON^ construct induced robust CD69 upregulation only in the presence of both rapalog and EGFR‐expressing target cells, whereas no T cell activation was detected in the absence of rapalog or in co‐cultures with EGFR‐negative target cells (Figure 5c). Consistent with these findings, rapalog‐dependent, antigen‐specific cytotoxicity was observed against EGFR‐positive HCT116 cells, whereas no cytotoxic activity was detected against EGFR‐negative CHO cells (Figure 5d). Collectively, these findings demonstrate that the TCE^ON^ platform can be readily adapted to alternative TAAs while preserving its inducible, rapalog‐dependent mechanism of action.

**FIGURE 5:**
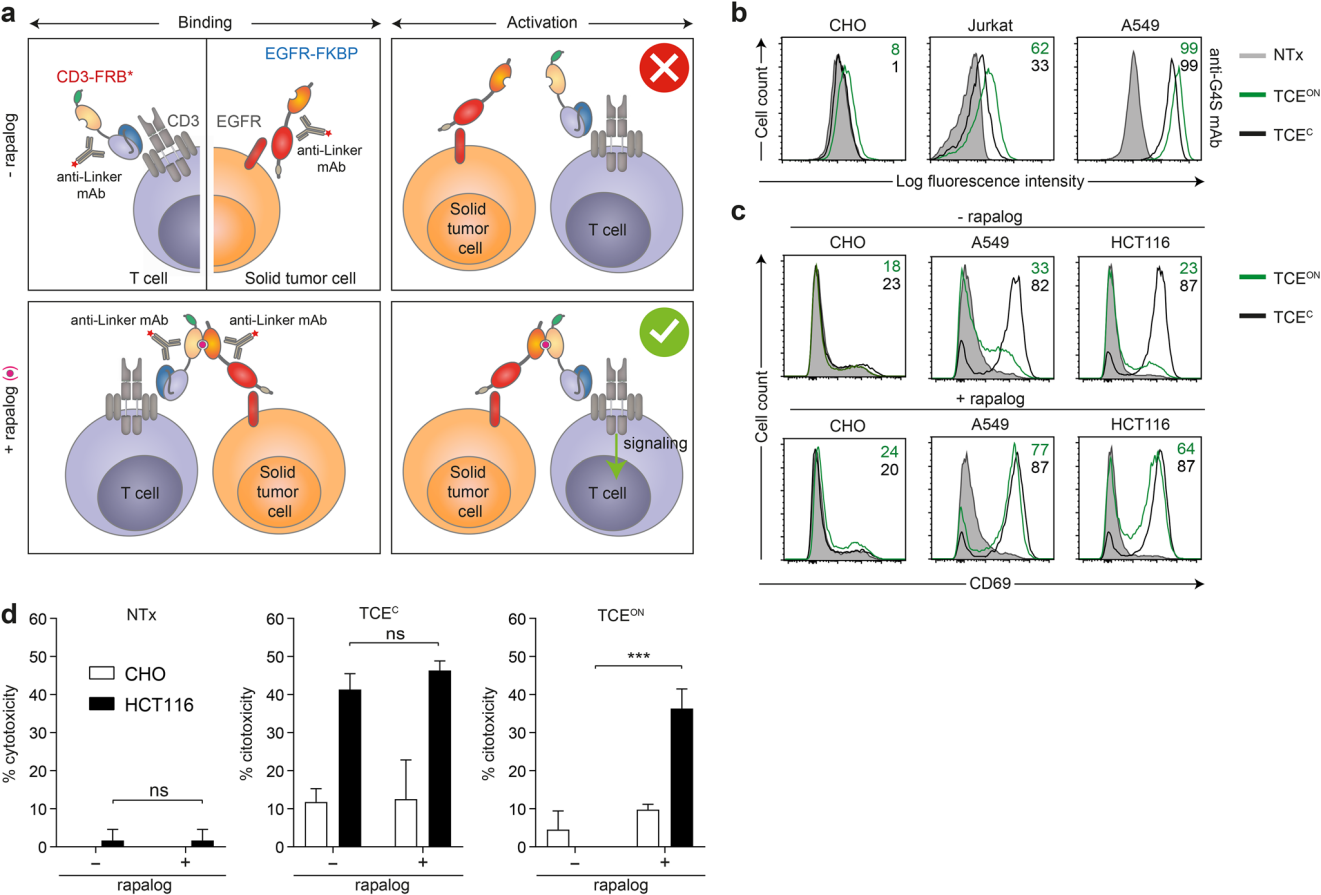
Functional characterization of an EGFR‐targeting TCE^ON^ construct. (a) Schematic illustration depicting cell binding (left) and the activation mechanism (right). (b) Detection of binding following antigen recognition, using an anti‐G4S linker mAb, for the TCE^ON^ modules (EGFR‐FKBP and CD3‐FRB*) present in CM from HEK293T cells transfected with the 2A‐based TCE^ON^ construct. (c) CD69 expression on primary T cells co‐cultured with CHO, A549, or HCT116 cells in the presence of CM from HEK293T cells transfected with the 2A‐based TCE^ON^ or TCE^C^ construct, with or without rapalog treatment. (d) Specific cytotoxicity assessed after 48 h of co‐culture of primary T cells with CHO^Luc^ or HCT116^Luc^ cells, in the presence of CM from NTx, TCE^C^, or TCE^ON^ transfected HEK293 cells, at 2:1 E:T ratio, with or without rapalog. Data are shown as mean ± SEM (*n* = 3); statistical significance was determined by two‐way ANOVA with Tukey's multiple comparisons test; ***p < 0.001. CM, conditioned media; NTx, non‐transfected.

## 4 Discussion

Redirecting T cells toward TAAs using bispecific TCEs or CARs has yielded substantial benefit, particularly in hematologic malignancies [49]. However, the absence of precise spatiotemporal control over T cell activity remains a major limitation, frequently resulting in dose‐limiting toxicities such as CRS, neurotoxicity, and on‐target/off‐tumor effects [50, 51]. Consequently, the development of controllable T cell platforms has emerged as a critical objective for improving the safety and adaptability of the cell based immunotherapies. Here, we present a novel, drug‐regulated STAb‐T cell platform based on a split CD19‐specific TCE (TCE^ON^) that enables tunable T cell activation through a clinically compatible small molecule, AP21967 (rapalog). Engineered T cells secrete two independent modules—CD19‐FKBP and CD3‐FRB*—that heterodimerize exclusively in the presence of rapalog to assemble a functional CD19 × CD3 bispecific antibody.

Although external control strategies for CAR‐T cells are rapidly evolving through advances in synthetic biology [52, 53, 54], comparable approaches for modulating TCE‐mediated T cell redirection remain unexplored. Our system builds upon the STAb‐T concept, which combines the trafficking and localization benefits of adoptively transferred T cells with the polyclonal T cell recruitment capabilities of secreted TCEs [2, 3]. The addition of a rapalog‐dependent molecular switch introduces an unprecedented level of control to this approach. Notably, both components of the TCE^ON^ were efficiently secreted by engineered cells, retained their target‐binding capacity, and dimerized in a dose‐dependent manner only upon drug induction, as shown by biochemical and functional assays. The rapalog‐induced dimerization translated into robust T cell activation and cytotoxicity specifically in the presence of CD19^+^ target cells, while avoiding activation in the absence of the drug. Interestingly, J‐STAb‐T^ON^ cells exhibited a higher proportion of activating immunological synapses than J‐STAb‐T^C^ cells, despite showing comparable F‐actin and pTyr accumulation at the synaptic interface. Although the mechanisms responsible for this difference remain unclear, it is conceivable that the modular architecture of the TCE^ON^ platform provides a degree of conformational adaptability that facilitates productive T cell–target cell engagement. Nevertheless, dedicated structural and biophysical studies will be required to directly evaluate this possibility.

The use of a 2A‐based bicistronic construct allowed efficient co‐expression of both TCE modules from a single vector, simplifying the generation of switchable STAb‐T^ON^ cells and enhancing their translational feasibility. Quantification of conditioned media demonstrated that both TCE^ON^ modules were secreted at comparable levels across the different producer cell types, with CD3‐FRB*/CD19‐FKBP ratios remaining close to unity. These findings indicate that the bicistronic construct supports near‐equimolar secretion of both modules while preserving efficient TCE assembly and functional activity. An important finding of this study is that the reduced IFN‐γ production by STAb‐T^ON^ cells is accompanied by a broader attenuation of pro‐inflammatory cytokine secretion, including TNF‐α, IL‐2, IL‐6, and GM‐CSF. This less inflammatory cytokine profile, together with the preservation of antigen‐specific cytotoxicity, suggests that TCE^ON^‐mediated activation may partially uncouple effector function from inflammatory cytokine production. Given that cytokine release syndrome remains one of the major toxicities associated with TCE therapies, these findings raise the possibility that pharmacologically control of TCE assembly could broaden the therapeutic window by limiting excessive cytokine release while maintaining antitumor activity.

Notably, our in vivo studies using xenograft models of CD19^+^ tumor cells provide compelling evidence of anti‐tumor efficacy. Mice receiving switchable STAb‐T^ON^ cells and rapalog showed significant tumor reduction, whereas mice treated with STAb‐T^ON^ cells without rapalog failed to show tumor control, confirming that therapeutic activity is strictly dependent on drug‐induced dimerization of the TCE modules. These findings demonstrate that the switchable system can elicit anti‐tumor responses in a temporally controlled manner, thereby offering an additional layer of safety by minimizing the risk of uncontrolled T cell activation. However, the anti‐tumor efficacy was not comparable to that observed with conventional STAb‐T^C^ cells, so further improvements are needed to balance the expression levels of the TCE modules or to improve the stability of the rapalog‐inducible TCE once formed. Since administration in our model was every 24 h, it is possible that interruptions in therapeutic activity may have occurred, allowing tumor proliferation, unlike the STAb‐T^C^ approach, which offers sustained production of TCE^C^. Conversely, this sustained TCE^C^ secretion, which in a clinical context could lead to increased toxicities, is tunable controlled by the exogenous administration of rapalog in the STAb^ON^‐T context. Although direct pharmacokinetic characterization of the assembled TCE^ON^ molecule was beyond the scope of this study, our findings indicate that rapalog availability is a key determinant of TCE^ON^ activity in vivo. The enhanced antitumor efficacy observed with the optimized dosing regimen, together with the loss of antitumor activity following rapalog withdrawal, demonstrates that therapeutic responses can be dynamically regulated through pharmacological control of TCE assembly. The reduced T cell persistence observed in the NT‐T group is consistent with the absence of antigen‐driven stimulation. In contrast, sustained persistence of STAb‐T cells, particularly under continuous rapalog administration, paralleled the improved antitumor responses and is consistent with ongoing rapalog‐dependent TCE^ON^ activity in vivo. The detection of circulating rapalog together with its stability after reconstitution further supports the feasibility of maintaining a biologically active dimerizer throughout the treatment period. Pharmacokinetic analysis showed that rapalog reached readily detectable plasma concentrations shortly after administration but declined rapidly within 24 h, indicating rapid systemic clearance rather than compound instability. Although circulating TCE bioactivity remained below the limit of quantification at the study endpoint, this observation is consistent with the low systemic exposure expected from a locally secreted engager [55]. Collectively, these findings indicate that the TCE^ON^ platform can achieve robust antitumor activity despite minimal systemic exposure to both the dimerizer and the secreted TCE. More broadly, they validate the central premise of the TCE^ON^ strategy, that T cell redirection can be dynamically and reversibly controlled through externally regulated assembly of the bispecific engager, and provide a pharmacokinetic rationale for the daily rapalog dosing schedule, including administration at the time of T cell infusion to ensure dimerizer availability during the critical early phase of TCE^ON^ assembly.

Our study positions the STAb‐T^ON^ platform as a versatile and controllable therapeutic strategy for CD19^+^ malignancies. However, an additional advantage of the TCE^ON^ platform is its potential applicability beyond hematological malignancies. Although the present study primarily employed the CD19 model to establish proof‐of‐concept and enable direct comparison with conventional STAb‐T cells, the modular nature of the system allows straightforward adaptation to alternative TAAs. In support of this concept, we demonstrate that an EGFR‐targeting TCE^ON^ construct retained rapalog‐dependent, antigen‐specific T cell activation and cytotoxicity, demonstrating that the strategy is readily adaptable to alternative TAAs. This feature may be particularly valuable in solid tumours, where on‐target/off‐tumour toxicity and systemic immune activation remain major limitations of TCE therapies [56]. In this context, local secretion of the split TCE modules, coupled with pharmacological control of their assembly, could provide an additional layer of spatial and temporal regulation, potentially reducing systemic exposure to the active TCE while preserving antitumour activity at the tumour site. Although these concepts require validation in clinically relevant solid tumour models, our findings suggest that TCE^ON^ may be especially well suited for solid tumours, where precise pharmacological control of T cell redirection could offer a broader therapeutic window.

However, a limitation of the present study is the absence of direct quantification of the secreted TCE within the tumor microenvironment. In contrast to systemically administered recombinant bispecific TCEs, whose biodistribution can be evaluated using radiolabeled or fluorescently labeled proteins [57], STAb‐T cells continuously secrete the TCE in situ, making direct assessment of its intratumoral distribution technically challenging. This limitation extends beyond our system: direct biochemical detection of a small‐molecule‐induced heterodimeric formation within tumor tissue in vivo has not been reported for other chemically regulated therapeutic systems, in which detection has been limited to the individual components in plasma [58] or to complex formation in cultured cells [59, 60]. Based on the established mechanism of action of bispecific TCEs, locally secreted molecules are expected to rapidly engage CD3‐positive T cells and tumor antigen‐expressing cells, resulting in their spatially restricted accumulation at sites of immune synapse formation. Consequently, bulk measurements in tumor lysates may underestimate the biologically active fraction and may not directly correlate with therapeutic efficacy. This observation is consistent with intravital imaging studies showing that the activity of bispecific TCE is governed by highly localized interactions between effector and target cells rather than solely by average tissue concentrations [61]. Future studies incorporating traceable STAb‐derived TCE constructs or dedicated molecular imaging approaches will be required to define the spatial distribution, local bioavailability, and spatiotemporal kinetics of in situ‐secreted TCEs in vivo.

It should also be acknowledged that the immunodeficient xenograft mouse models used in this study do not fully recapitulate the complexity of the human immune system and therefore have limited predictive value for CRS and other immune‐mediated toxicities. Although the reduced cytokine secretion observed in vitro suggests that pharmacological control of TCE assembly may mitigate such toxicities, this hypothesis will require validation in more physiologically relevant preclinical models. Future studies using humanized mouse models or other advanced experimental systems will be necessary to characterize cytokine‐mediated toxicities more comprehensively and evaluate the translational safety of the inducible TCE^ON^ platform.

The EGFR‐directed proof‐of‐concept construct demonstrates that the inducible TCE^ON^ architecture can be readily redirected toward a second, unrelated tumor antigen while preserving rapalog‐dependent activity, supporting the modularity of the platform. Future studies will be required to validate additional tumor‐targeting modules in vivo using appropriately selected solid tumor models. Beyond antigen redirection, the modular architecture of TCE^ON^ provides a foundation for developing more sophisticated multi‐input regulatory circuits, including AND‐ and OR‐gated systems, to further enhance the precision and selectivity of T cell redirection. Comprehensive assessment of long‐term safety and the potential immunogenicity of the split components will also be essential to support the clinical translation of this platform.

## 5 Conclusions

Collectively, our results demonstrate the feasibility of engineering switchable STAb‐T^ON^ cells controlled by a drug‐inducible TCE for the treatment of CD19^+^ tumors. These findings represent a promising advancement in the field of cell‐based immunotherapy, which could be adapted for other hematological or solid malignancies, while offering a treatment with enhanced safety, tunability, and therapeutic control after administration.

## Author Contributions

Conception and design of the study: A.J.‐R. and L.A.‐V. Acquisition of data. S.L.‐A., C.D.‐A., I.Z., A.T.‐G., E.G.‐V., L.R.‐P., M.G.‐R., P.F., P.H.‐L., O.A.‐S., L.A.‐P., S.M.‐G., L.C., M.R.‐S., B.B., A.R.‐F., L.D.‐A., J.A.‐R., A.J.‐R. Analysis and interpretation of data: S.L.‐A., C.D.‐A., I.Z., A.T.‐G., E.G.‐V., P.F., P.H.‐L., O.A.‐S., S.M.‐G., L.A.‐P., J.M.‐T., M.L.T., C.B.‐A., P.R.‐N., A.J.‐R., and L.A.‐V. Writing – original draft: C.D.‐A., S.L.‐A., A.J.‐R. and L.A.‐V. Review and editing: All authors. Guarantors: A.J.‐R. and L.A.‐V. All authors read and approved the final manuscript.

## Funding

Research in LA‐V laboratory is funded by the MCIN/AEI/10.13039/501100011033 (PID2023‐148429OB‐I00, PID2020‐117323RB‐I00, PDC2021‐121711‐ I00, CPP2022‐009762, CPP2022‐009765, CPP2023‐010827), the Carlos III Health Institute (ISCIII) with European Regional Development Fund (FEDER) cofinancing (DTS20/00089, PMPTA22/00167); the Asociación Española contra el Cáncer (AECC) (PROYE19084ALVA and PRYGN234844ALVA), the CRIS Cancer Foundation (FCRIS‐ 2021‐0090 and FCRIS‐2023‐0070), the Fundación “La Caixa” (HR21‐00761 project IL7R_LungCan), the Fundación para la Investigación Biomédica del Hospital 12 de Octubre (programa Investiga 2022‐0082) and the Fundación Fero (BBASELGAFERO2024.01). AJ‐R is supported by the ISCIII (PI23/01256) and the Comunidad de Madrid (P2022/BMD‐7225 Next Generation CART MAD). PR‐N is funded by MCIN/AEI/10.13039/501100011033 (PID2020‐115444GB‐I00 and PID2023‐147805NB‐I00). MLT is supported by MCIN/AEI/10.13039/501100011033 (PID2019‐105623RB‐I00) and the AECC (CICPF18030TORI). BB is supported by the ISCIII (PI23/01256), AECC (INNOV211832BLAN) and Comunidad de Madrid (IND2022/BMD‐23732). SL‐A is supported by the Comunidad de Madrid (P2022/BMD‐7225 Next Generation CART MAD). CD‐A was supported by a predoctoral fellowship from the MCIN/AEI/10.13039/501100011033 (PRE2018‐083445). LR‐P is supported by a predoctoral fellowship from the Immunology Chair, Universidad Francisco de Vitoria/Merck. MG‐R is supported by an Industrial PhD fellowship from Comunidad de Madrid (IND2022/BMD‐23732). OA‐S was supported by a PhD fellowship from the Complutense University of Madrid. LD‐A was supported by a Rio Hortega fellowship from the ISCIII (CM20/00004).

## Ethics Approval and Consent to Participate

All samples were obtained after written informed consent from the donors and all studies were performed according to the principles expressed in the Declaration of Helsinki and approved by the Institutional Research Ethics Committees of the hospitals and research centers involved. All the in vivo experiments were carried out in accordance with the European Union Directive 2010/63/UE, enforced in Spanish law under RD 53/2013. All animal experiments were approved by the respective Ethics Committees of Animal Experimentation of the Madrid Community and Instituto de Salud Carlos III; they were performed in accordance with the guidelines stated in the International Guiding Principles for Biomedical Research Involving Animals, established by the Council for International Organizations of Medical Sciences. The experimental study protocols were additionally approved by local government (PROEX 272.2/23)

## Conflicts of Interest

LA‐V and BB are co‐founders and shareholders of STAb Therapeutics, a spin‐off company from the imas12. LA‐V and BB are inventors on the patent EP21708942 pending. LA‐V and LD‐A are inventors on the patent EP23383410.0 pending. LA‐V is co‐founder and shareholder of Trimerbody Pharma, a company focused on unrelated interest. LA‐V reports speaker honoraria from MSD, Merck KGaA, BMS, Janssen, GSK, and Miltenyi, and receives grant support from Merck KGaA, all outside the submitted work.

## Supporting information




**Supporting File**: advs77236‐sup‐0001‐SuppMat.pdf.

## Data Availability

All data relevant to the study are included in the article or uploaded as supplementary information. Further information and requests for resources and reagents should be directed to and will be fulfilled by Luis Álvarez‐Vallina (lalvarezv@ext.cnio.es). Plasmids generated in this study will be made available on request, but we may require a completed Material Transfer Agreement if there is potential for commercial application. This paper does not report original code.
