# Supplementary material for: A Drug‐Gated, Modular STAb‐T Immunotherapy With External Control
Source: Adv Sci (Weinh). 2026 Aug 24;13(61):e77236. doi: 10.1002/advs.77236 (PMC13502364; doi:10.1002/advs.77236)
Supplement: Supplementary file 1 [file ADVS-13-e77236-s001.pdf]

Inventory of Supporting Information for

A drug-gated, modular STAb-T immunotherapy with external control

Susana Luengo-Arias[#], Carmen Domínguez-Alonso[#], Ivana Zagorac, Antonio Tapia-Galisteo, Eva García-Veros, Laura Rubio-Pérez, Marina Gómez-Rosel, Patricia Fuentes, Patricia Hernández-López, Óscar Aguilar-Sopeña, Laura Almagro-Puente, Sonia Martínez-González, Lucia Cañizares, Maria Rivas-Sanchez, Belén Blanco, Ángel Ramírez-Fernández, Laura Díez-Alonso, Carmen Blanco-Aparicio, Javier Arroyo-Ródenas, Jorge Martínez-Torrecuadrada, María L. Toribio, Pedro Roda-Navarro, Anaïs Jiménez-Reinoso[*], Luis Álvarez-Vallina[*]

[#]These authors contributed equally

***Corresponding authors:** a.jimenez.imas12@h12o.es, lalvarezv@ext.cnio.es

This Inventory includes:

- **Supplementary Figures**
- **Supplementary Tables**
- **List of abbreviations**

SUPPLEMENTARY FIGURES

Supplementary Figure 1

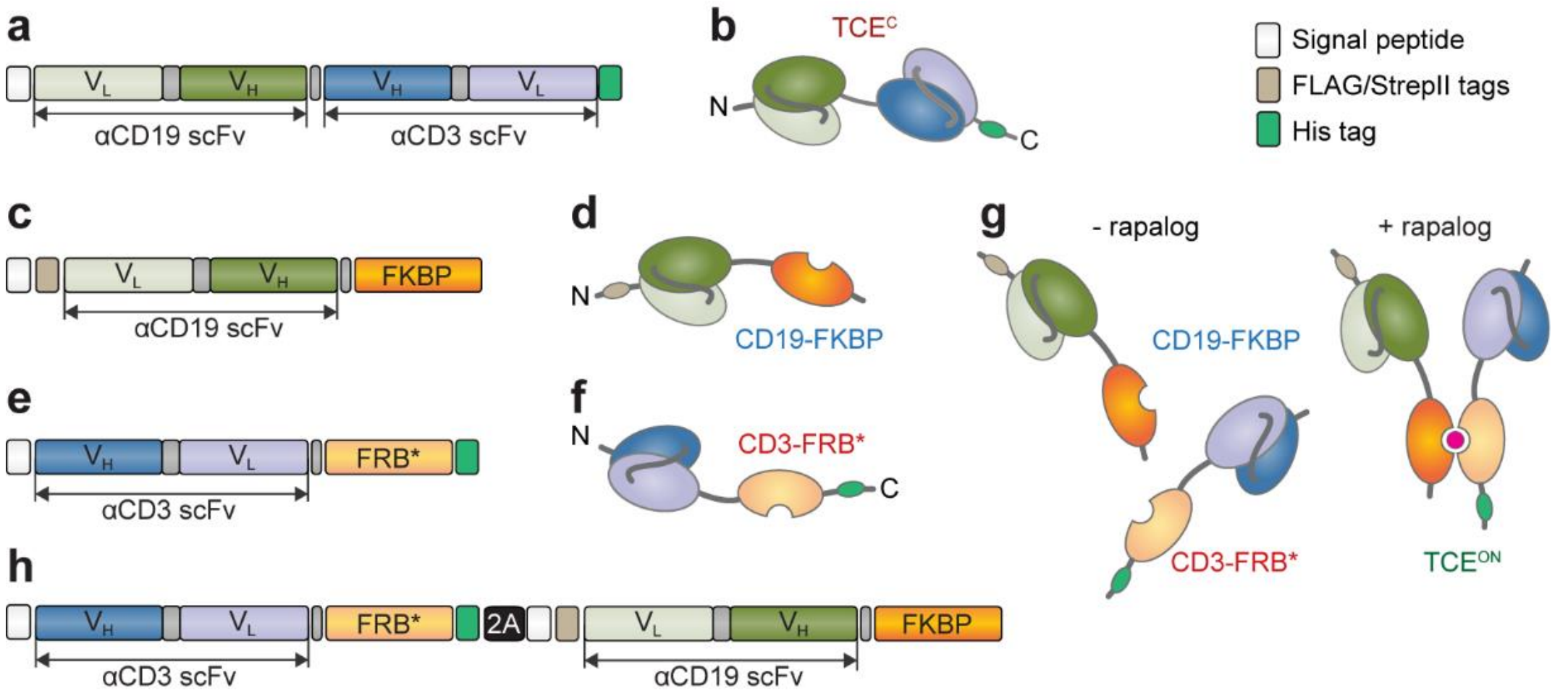

Figure S1. Schematic representations of the genic constructs and and domain organization of the TCE^{C} and TCE^{ON} modules (CD19-FKBP and CD3-FRB*). (a, b) CD19-TCE^{C} (TCE^{C}) is formed by an anti-CD19 A3B1 scFv gene (green boxes) fused to an anti-CD3 OKT3 scFv gene (blue boxes) and includes a C-terminal His tag (teal box). **(c, d)** The tumor antigen recognition module of CD19-TCE^{ON} (CD19-FKBP) consists of an anti-CD19 scFv bearing N-terminal FLAG and StrepII tags (grey box), followed by an FKBP dimerization domain (dark orange box). **(e, f)** The T cell recognition module of the CD19-TCE^{ON} (CD3-FRB*) contains an anti-CD3 scFv fused to a FRB* domain (light orange box) with a C-terminal His tag. TCE^{C}, CD19-FKBP, and CD3-FRB* are each preceded by an N-terminal oncostatin M signal peptide (white box), directing secretion of the encoded proteins. (**g**) Conformations of the CD19-TCE^{ON} modules in the absence or presence of rapalog. FKBP and FRB* domains heterodimerize only in the presence of rapalog, allowing the composition of a bispecific TCE antibody with simultaneous recognition of the tumor antigen and CD3 on T cells. (**h**) Genetic construct of the 2A-based bicistronic cassette for the coordinated expression of the CD3-FRB* and CD19-FKBP modules.

Supplementary Figure 2

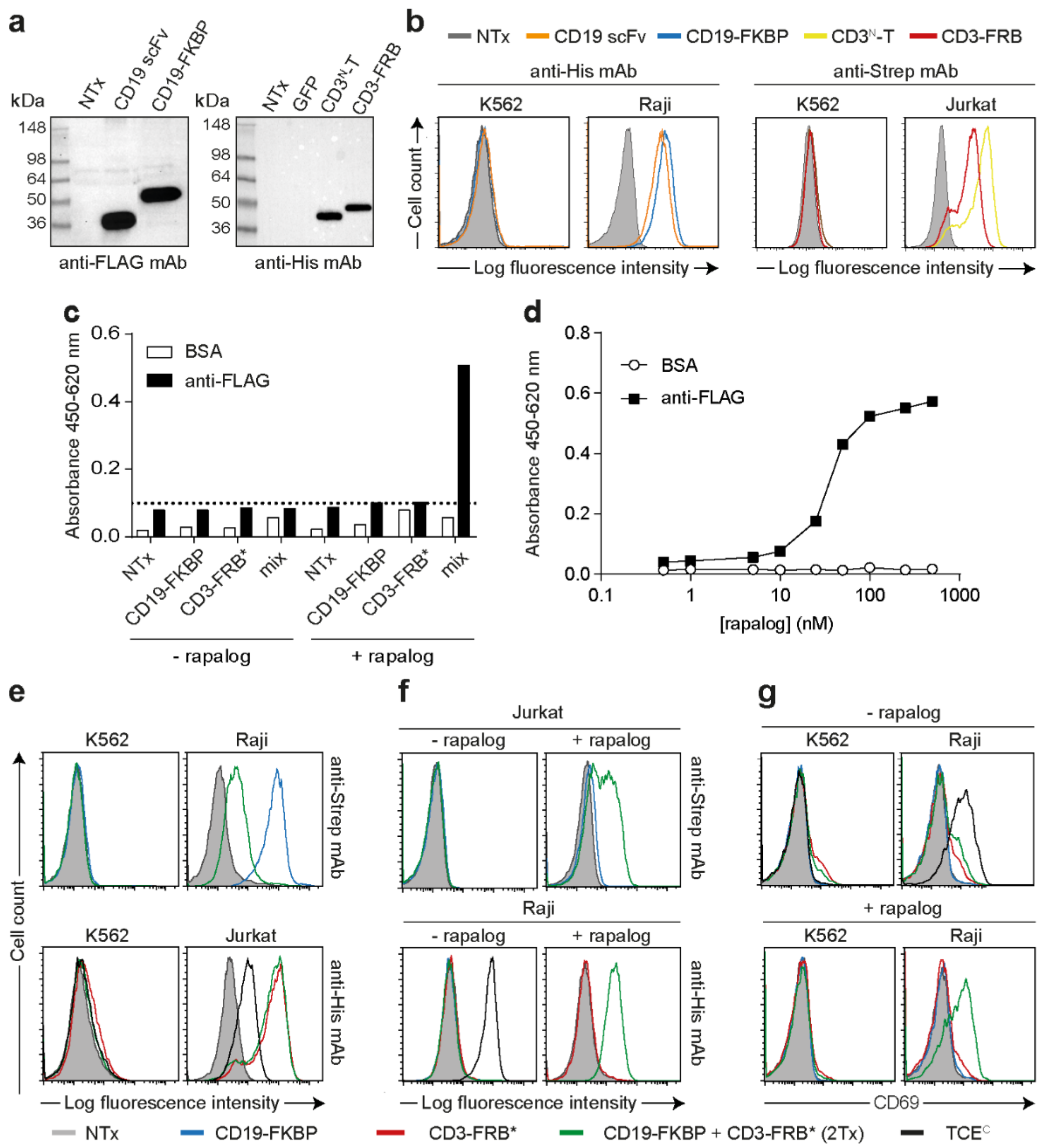

Figure S2. Characterization of the CD19-TCE^{ON} (a) Detection of soluble CD19-FKBP or CD3-FRB* from the CM of transiently transfected HEK293 cells by WB. Blots were developed with an anti-FLAG or an anti-His mAb, to detect CD19-FKBP or CD3-FRB*, respectively. **(b)** Binding analysis of the secreted CD19-FKBP and CD3-FRB* to its corresponding antigens in a cellular context. CM from NTx or transfected HEK293 cells with GFP, CD19 scFv or CD3^{N}-T were used as negative or positive controls in **(a, b)**. **(c)** Detection of dimer formation between CD19-FKBP and CD3-FRB* when rapalog is added to a mix of CM from CD19-FKBP and CD3-FRB*-transfected HEK293 cells by ELISA using immobilized anti-FLAG or BSA. **(d)** Rapalog-dependent kinetics of CD19-FKBP:CD3-FRB* formation by ELISA using immobilized anti-FLAG or BSA. **(e)** Detection of the presence and antigen recognition of the modules of the TCE^{ON} in CM

from mono- or double-transfected (2Tx) HEK293 cells. **(f)** Detection of the formed dimer in CM from rapalog-treated 2tx HEK293 cells. CM from NTx or mono-transfected HEK293 was used as control. **(g)** CD69 expression in primary T cells after co-culture with K562 or Raji cells in presence of CM from NTx, 2tx or mono-transfected HEK293 cells, treated or not with rapalog. CM, conditioned media; WB, Western blot; NTx, non-transfected, 2tx, double-transfected.

Supplementary Figure 3

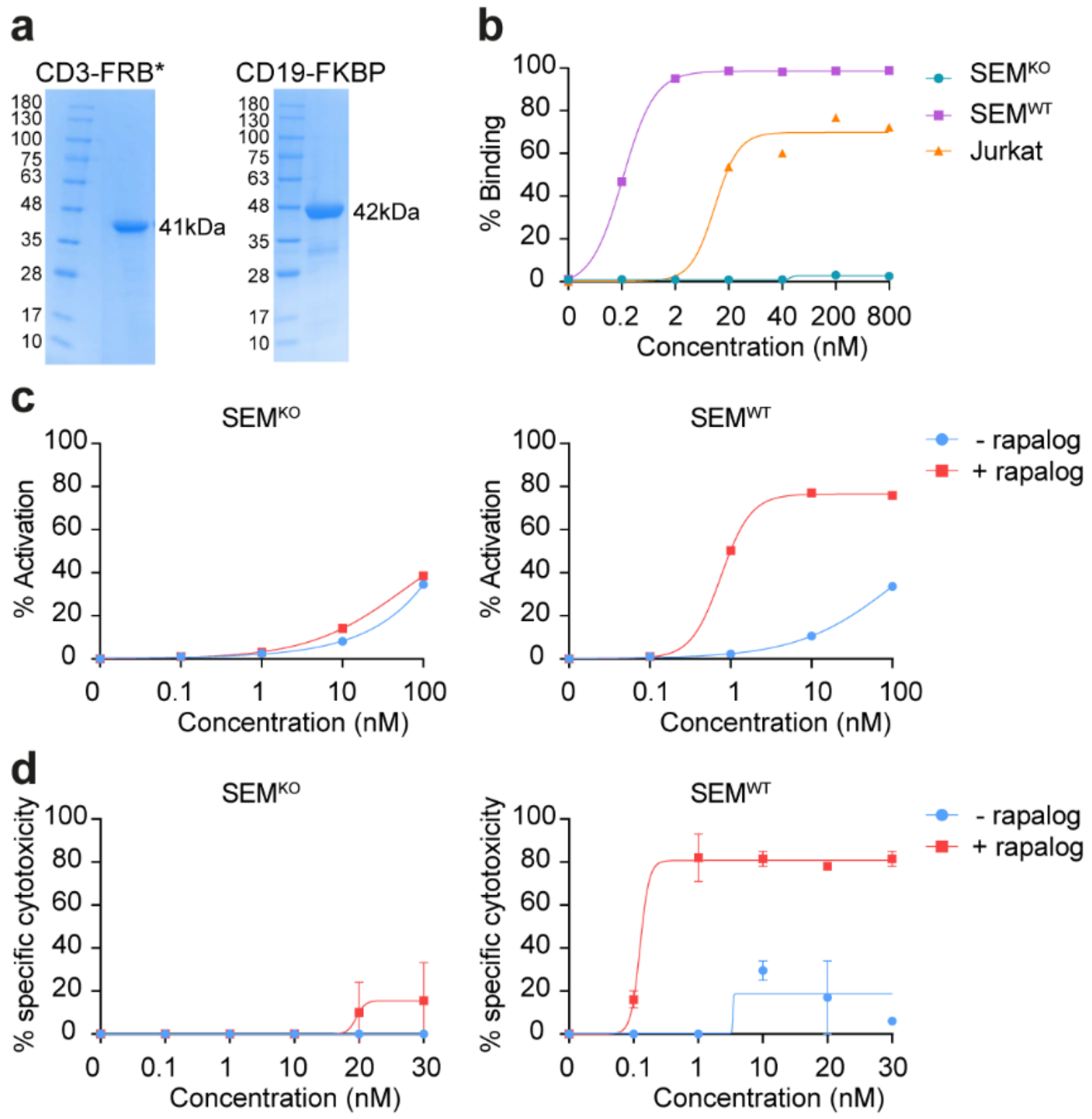

Figure S3. Functional characterization of purified CD3–FRB* and CD19–FKBP. (a) Coomassie-stained SDS–PAGE demonstrating purified CD3–FRB* and CD19–FKBP. Molecular-weight markers (kDa) are indicated. **(b)** Flow cytometry analysis of binding between purified CD3–FRB* and Jurkat cells, and between CD19–FKBP and SEM^{WT} or SEM^{KO} cells, across concentrations ranging from 0–800 nM. **(c)** CD69 upregulation in Jurkat (JK) cells after 24 h co-culture with SEM^{KO} (left) or SEM^{WT} (right) (right) cells in the presence of increasing concentrations (0–100 nM) of CD3–FRB* and CD19–FKBP, together with 500 nM rapalog, quantified by flow cytometry. **(d)** Antigen-specific cytotoxicity of primary T cells co-cultured for 48 h with SEM^{KO} (left) or SEM^{WT} (right) cells in the presence of increasing concentrations (0–30 nM) of CD3–FRB* and CD19–FKBP, plus 500 nM rapalog, as determined by luminescence.

Supplementary Figure 4

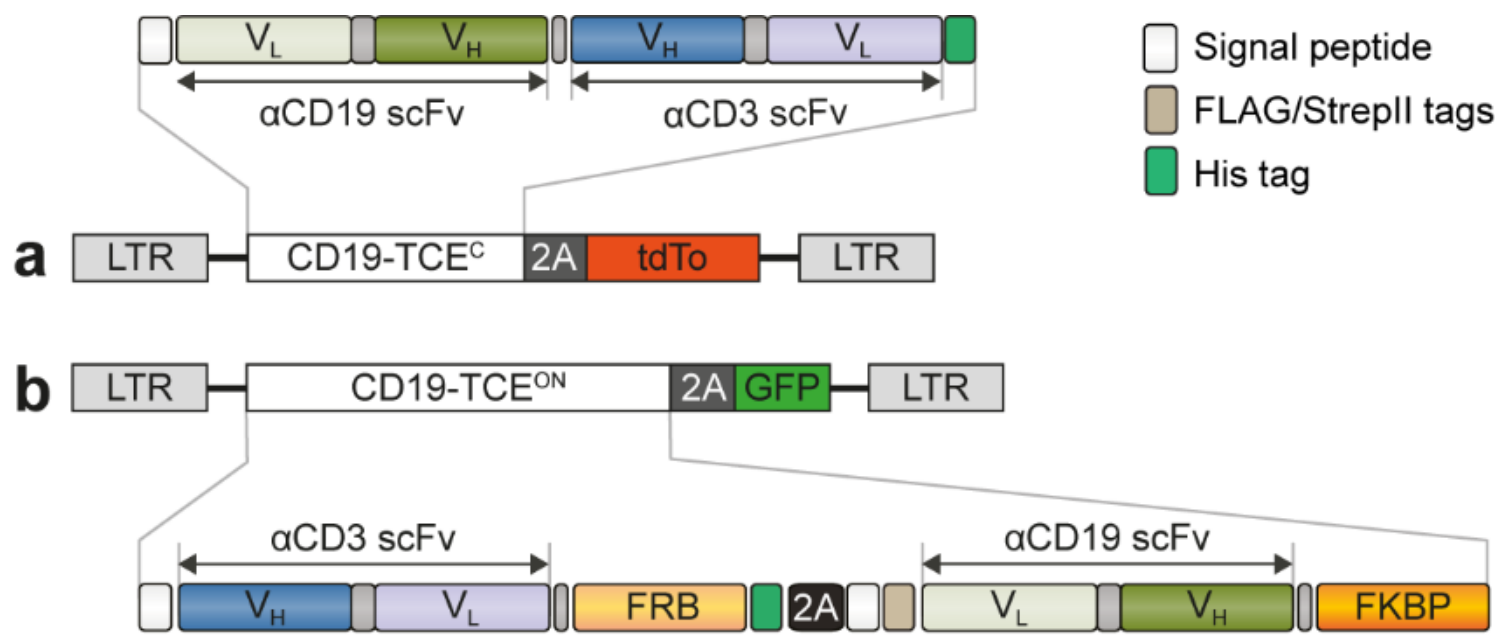

Figure S4. Schematic representation of the 2A-based lentiviral expression vectors. **(a)** Conventional TCE^{C} construct encoding an anti-CD19scFv-anti-CD3scFv bispecific TCE with a C-terminal His tag (teal box), followed by tdTomato (tdTo) via a *Thosea asigna* virus 2A peptide. **(b)** TCE^{ON} construct encoding an anti-CD3scFv-FRB*-His tag module a FLAG-StrepII tag (grey box) anti-CD19scFv-FKBP module, separated by a *Thosea asigna* virus 2A peptide, with GFP expressed from a second downstream *Thosea asigna* virus 2A peptide. Oncostatin M signal peptides are indicated by white boxes.

Supplementary Figure 5

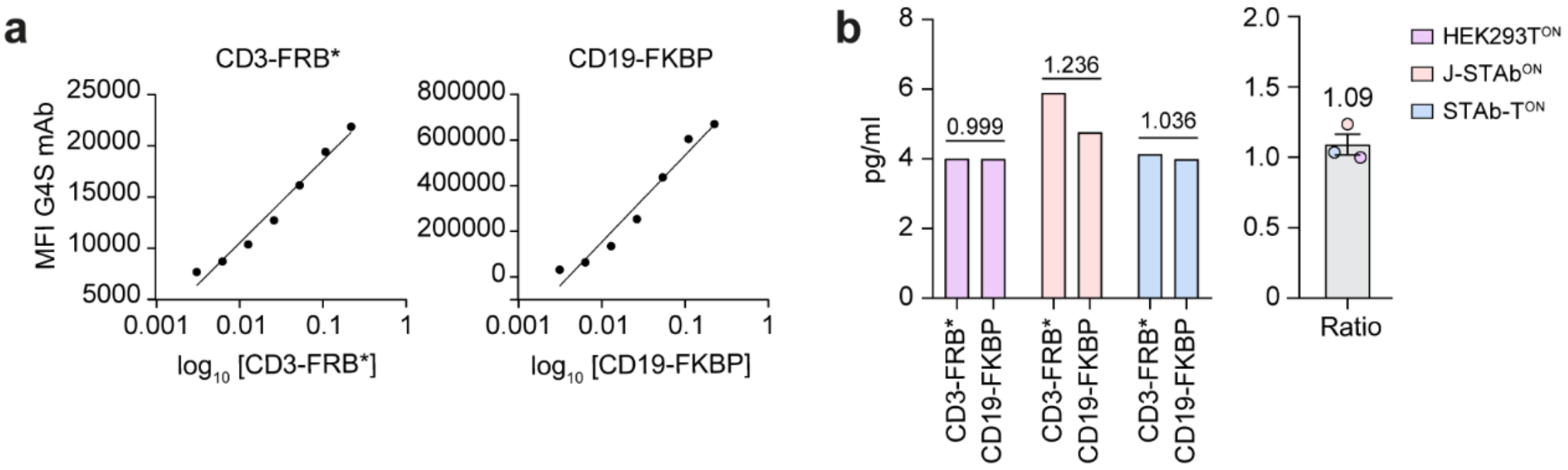

Figure S5. Quantification of CD3-FRB* and CD19-FKBP modules in conditioned media from TCE^{ON}-producing cells. (a) Standard curves were generated using purified CD3 and CD19 proteins via binding to Jurkat cells (for CD3) and SEM^{WT} cells (for CD19), respectively (Curve CD3, Curve CD19). Linear regression analysis yielded R^2 values of 0.9742 and 0.9612 for the CD3 and CD19 standard curves, respectively. **(b)** The same binding assay was performed using conditioned media from HEK293T cells transfected with the 2A-based TCE^{ON} construct (HEK293T^{ON}), from transduced Jurkat cells (J-STAb^{ON}), and from transduced T cells (STAb-T^{ON}). In all cases, each module was detected by binding to Jurkat or SEM^{WT} cells followed by staining with an anti-G4S linker mAb, and the corresponding concentrations were extrapolated from the respective standard curve. The CD3-FRB*/CD19-FKBP ratio was calculated for each sample from the individually extrapolated module concentrations, and the mean ratio across samples is shown in the right.

Supplementary Figure 6

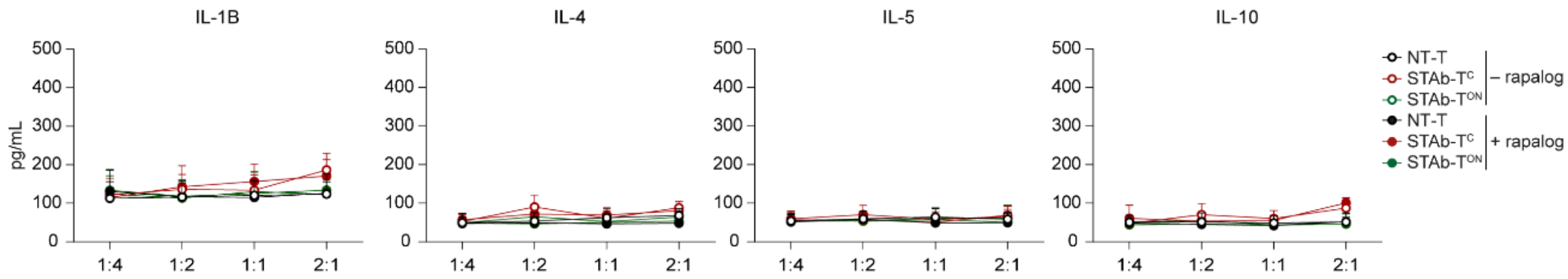

Figure S6. Anti-tumor activity *in vitro* of inducible primary STAb-T^{ON} T cells (cont.). Pro- and anti-inflammatory secretion profile quantified by multiplex bead-based immunoassay (IL-1β, IL-4, IL-5, and IL-10) in supernatants collected from the cytotoxicity assay described in Fig. 3, performed at the indicated E:T ratios.

Supplementary Figure 7

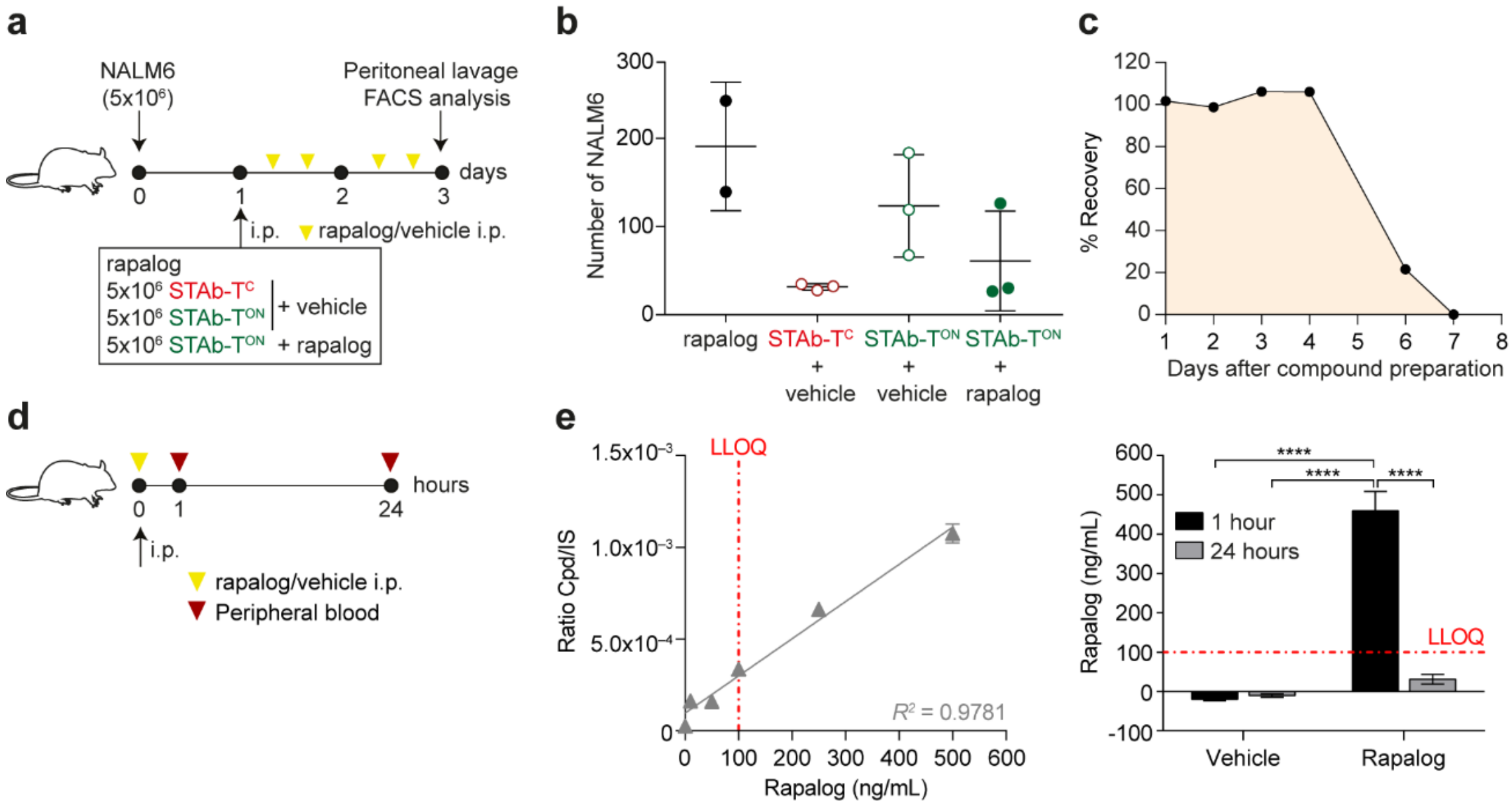

Figure S7. Anti-tumor activity *in vivo* of inducible STAb-T^{ON} T cells. (a) Short-term *in vivo* experiment in NSG mice. Mice (n=3 per group) were intraperitoneally (i.p). injected with 5×10^{6} NALM6 cells, and 24 hours later, with rapalog or with 5×10^{6} T cells (NT-T, STAb-T^{C} or STAb-T^{ON}) plus rapalog or vehicle. Subsequent treatments with rapalog or vehicle were administered as indicated. **(b)** Mice were sacrificed 48 hours later, and peritoneal lavage was analyzed for the detection of CD19^{+} NALM6 by flow cytometry. **(c)** Stability of rapalog in the formulation used for *in vivo* administration (16.7% 1,2-propanediol, 22.5% PEG 400, and 1.25% Tween 80), expressed as percentage of compound recovery over time after preparation. Samples were analyzed by reversed-phase HPLC-UV, and compound concentrations were determined from the chromatographic peak area using a calibration curve. Recovery remained close to 100% up to day 4, followed by a marked decline thereafter. Data in **(b)** represent mean ± SEM and statistical significance was calculated by one-way ANOVA test corrected by Tukey's multiple comparisons test. **(d)** Experimental design of the plasma pharmacokinetic study. Mice received a single i.p. injection of rapalog or vehicle at time 0. Peripheral blood was collected 1 hour and 24 hours after administration for plasma isolation and rapalog quantification. **(e)** Plasma pharmacokinetics of rapalog following a single i.p. injection. **Left**, representative calibration curve for rapalog quantification by HPLC-MS/MS. Calibration standards were prepared in mouse plasma, analyzed in technical triplicate, and are presented as mean ± SD. The calibration curve was fitted by linear regression (R^2 = 0.9781). The red dashed line indicates the lower limit of quantification (LLOQ). **Right**,

plasma rapalog concentrations measured 1 hour and 24 hours after a single i.p. injection of rapalog or vehicle. Data are shown as mean ± SEM (n = 3 mice per group; technical triplicates). Statistical significance was determined by two-way ANOVA followed by Tukey's multiple comparisons test. ****$P < 0.0001$; i.p., intraperitoneally.

Supplementary Figure 8

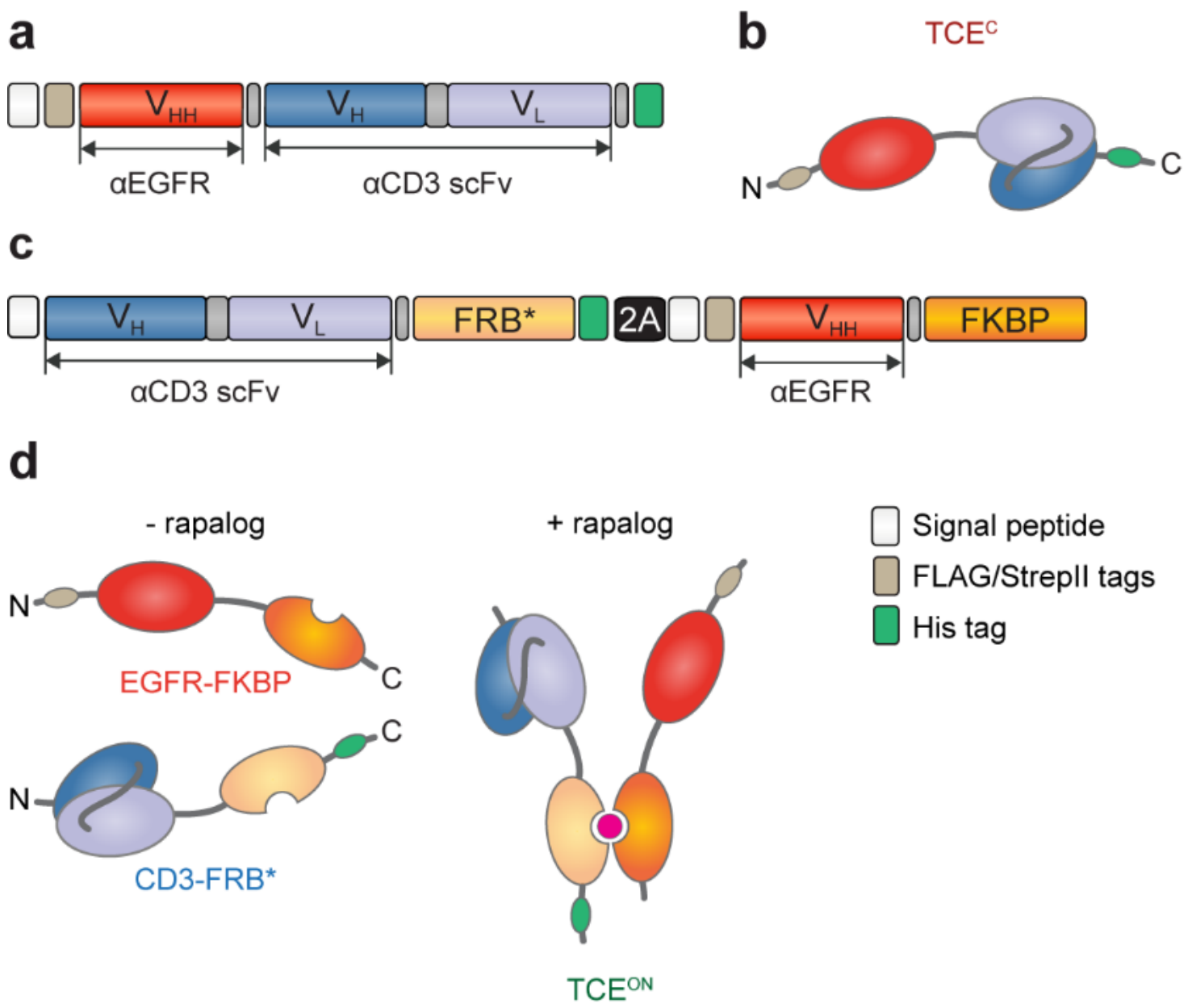

Figure S8. Schematic representations of the genic constructs and protein structures of the TCE^{C} and TCE^{ON} modules (EGFR-FKBP and CD3-FRB*). (a, b) EGFR-TCE is formed by an anti-EGFR scFv gene (red boxes) fused to an anti-CD3 OKT3 scFv gene (blue boxes) and includes a C-terminal His tag (teal box). **(c)** Genetic construct of the 2A-based bicistronic cassette for simultaneous expression of CD3-FRB* and EGFR-FKBP modules. **(d)** EGFR-TCE^{ON} module conformations in the absence or presence of rapalog, showing dimerization of the FKBP and FRB* domains only in the presence of rapalog.

SUPPLEMENTARY TABLES

Supplementary table S1. Antibodies and reagents

Antibodies

Antigen	Clone	Host	Brand	Reference	Application
Anti-human CD2 APC-H7	RPA.2.10	mouse	BD Biosciences	562638	FC
Anti-human CD3 APC	UCHT-1	mouse	BD Biosciences	555335	FC
Anti-human CD3 APC	UCHT-1	mouse	BD Biosciences	555335	FC
Anti-human CD3 PC7	UCHT-1	mouse	BD Biosciences	563423	FC
Anti-human CD3 V450	UCHT-1	mouse	BD Biosciences	560366	FC
Anti-human CD4 PE	L200	mouse	BD Biosciences	555347	FC
Anti-human CD8 PC7	RPA-T8	mouse	BD Biosciences	560662	FC
Anti-human CD10 APC	HI10a	mouse	BD Biosciences	340923	FC
Anti-human CD10 APC/Cy7	HI10a	mouse	Biolegend	312220	FC
Anti-human CD19 PC7	J3.119	mouse	Beckman Coulter	IM3628	FC
Anti-human CD45 V450	HI30	mouse	BD Biosciences	560367	FC
Anti-human CD69 APC	L78	mouse	BD Biosciences	560689	FC
Anti-human CD69 PC7	L78	mouse	BD Biosciences	560819	FC
Anti-human CD69 PE	L78	mouse	BD Biosciences	555531	FC
Anti-human G4S linker	E702V	rabbit	Cell signaling	69782L	FC
Anti-human HLA-ABC APC	G46-2.6	mouse	BD Biosciences	562006	FC
FLAG HRP	M2	mouse	Sigma-Aldrich	A8592	ELISA, WB
FLAG tag	M2	mouse	Sigma-Aldrich	A8592	WB, ELISA
IgG mouse AF®647	Policlonal	goat	Invitrogen	A21235	FC
IgG mouse PE	Policlonal	goat	Jackson	115-116-146	FC
IgG rabbit AF®488	Policlonal	donkey	Life Technologies	A21206	IHC
IgG rabbit AF®594	Policlonal	goat	Life Technologies	A11012	IHC
Poli-His HRP	His-1	mouse	Sigma-Aldrich	A7058	ELISA, WB
Poli-His tag	PentaHis	mouse	QIAgen	34660	WB
Poli-His tag Biotin	HIS.H8	mouse	Invitrogen	MA1-21315-BTIN	FC
pTyr	P-Tyr-1000	rabbit	Cell signaling	8954	IHC
Strep-tagII		mouse	IBA Biosciences	2-1507-001	FC, WB

Reagents

Compound	Brand	Reference
7-ADD	BD Biosciencies	559925
A/C Heterodimerizer (rapalog *in vitro*)	Takara	635056
A/C Heterodimerizer (rapalog *in vivo*)	Takara	635055
CellTrace Far Red	Invitrogen	C34564
CellTrace Violet	Invitrogen	C345578
CellTracker Blue (CMAC)	Molecular Probes	C211013410
Cytokine Human Magnetic 10-Plex Panel for Luminex™ Platform	ThermoFisher Scientific	LHC0001M
Corning® Matrigel®	Corning	356231
D-luciferin	Promega	E1605
DAPI	Sigma-Aldrich	D9542

Dynabeads™ Human T-Activator CD3/CD28	ThermoFisher Scientific	11131D
Human Dissociation Kit	Miltenyi Biotec	130-095-929
Human IFN-γ ELISA Kit	Diaclone	950-090-096
Lipofectamine™ 3000 Reagent Kit	Invitrogen	L3000015
Lymphoprep	Stem Cell Technologies	7851
Mycoplasma Gel Detection Kit	Biotools	90.022-4544
Pan T-Cell Isolation Kit	Miltenyi Biotec	130-096-535
Phalloidin AF®647	Molecular Probes	A2228711
Streptavidin APC	Molecular Probes	SA1005
Streptavidin PE	BD Biosciences	554061

IHC, Immunohistochemistry; FC, Flow Cytometry; ELISA, Enzyme-linked immunosorbent assays; WB, Western blot.

LIST OF ABBREVIATIONS

2Tx: double transfected HEK293 cells (CD19-FKBP+CD3-FRB*)

Blue CMAC: 7-amino-4-Chloromethylcoumarin

CAR: Chimeric antigen receptor

CM: Conditioned media

CRS: Cytokine release syndrome

DCM: DMEM complete medium

E:T: Effector:target

EGFR: epidermal growth factor receptor

FITC: Fluorescein isothiocyanate

FKBP: FK506 binding protein

FRB*: FKBP rapamycin binding domain (T2089L)

F/S: FLAG and Strep-tagII epitopes

GFP: Green fluorescent protein

G-418: Geneticin

HEK293T^{ON}: HEK293T cells secreting TCE^{ON}

HPLC: High-Performance Liquid Chromatography

HRP: Horseradish peroxidase

IFN-γ: Interferon-γ

IL: Interleukin

IMAC: Immobilized-metal affinity chromatography

i.p.: Intraperitoneally

IS: Immunological synapse

J-NT: Non-transduced Jurkat T cells

J-STAb-T^{C}: Jurkat STAb-T cells secreting TCE^{C}

J-STAb-T^{ON}: Jurkat STAb-T cells secreting TCE^{ON}

KO: Knock-out

LLOQ: Lower Limit of Quantification

Luc: Firefly luciferase gene

mAb: Monoclonal antibodies

MFI: median fluorescence intensity

MS: mass spectrometry

MOI: Multiplicities of infection

NT-T: Non-transduced primary T cells

NTx: Non-transfected cells

NSG: NOD.Cγ-*Prkdc*^{scid} *Il2rg*^{tm1Wjl}/SzJ mice female mice

NXG: NOD-*Prkdc*^{scid}-*IL2rg*^{Tm1}/Rj female mice

OM: Oncostatin M

PBMC: Peripheral blood mononuclear cells

PROTAC: Proteolysis-targeted chimera

pTyr: Phospho-tyrosine

RCM: RPMI complete medium

Rapalog: Rapamycin analog

s.c.: Subcutaneously

scFv: Single-chain variable fragment

SD: Standard deviation

SEC: Size-exclusion chromatography

SEE: Staphylococcal enterotoxin E

SEM: Standard error of the mean

STAb: Secreting T cell-engaging antibodies

STAb-T^{C}: STAb-T cells secreting TCE^{C}

STAb-T^{ON}: STAb-T cells secreting TCE^{ON}

TAA: Tumor-associated antigens

TCEs: T cell engagers

TCE^{C}: Conventional anti-CD19 TCE

TCE^{ON}: ON-switch anti-CD19 TCE

T_{CM}: Central memory T cells

T_{EF}: Effector T cells

T_{EMRA}: Effector memory T cells expressing CD45RA

tdTo: tdTomato fluorescent protein

TMB: Tetramethylbenzidine

T_{N}: Naïve T cells

UV: Ultraviolet

V_{H}: Variable heavy

V_{L}: Variable light
